# Local microRNA delivery targets Palladin and prevents metastatic breast cancer

**DOI:** 10.1038/ncomms12868

**Published:** 2016-09-19

**Authors:** Avital Gilam, João Conde, Daphna Weissglas-Volkov, Nuria Oliva, Eitan Friedman, Natalie Artzi, Noam Shomron

**Affiliations:** 1 Department of Cell and Developmental Biology, Sackler Faculty of Medicine, Tel-Aviv University, Tel-Aviv 69978, Israel; 2 Massachusetts Institute of Technology, Institute for Medical Engineering and Science, Cambridge, Massachusetts 02139, USA; 3 School of Engineering and Materials Science, Queen Mary University of London, London E1 4NS, UK; 4 The Susanne Levy Gertner Oncogenetics Unit, The Danek Gertner Institute of Human Genetics, Chaim Sheba Medical Center Tel-Hashomer, 52621 Ramat Gan, Israel; 5 Broad Institute of MIT and Harvard, Cambridge, Massachusetts 02142, USA; 6 Department of Medicine, Biomedical Engineering Division, Brigham and Women's Hospital, Harvard Medical School, Boston, Massachusetts 02115, USA

## Abstract

Metastasis is the primary cause for mortality in breast cancer. MicroRNAs, gene expression master regulators, constitute an attractive candidate to control metastasis. Here we show that breast cancer metastasis can be prevented by miR-96 or miR-182 treatment, and decipher the mechanism of action. We found that miR-96/miR-182 downregulate Palladin protein levels, thereby reducing breast cancer cell migration and invasion. A common SNP, rs1071738, at the miR-96/miR-182-binding site within the Palladin 3′-UTR abolishes miRNA:mRNA binding, thus diminishing Palladin regulation by these miRNAs. Regulation is successfully restored by applying complimentary miRNAs. A hydrogel-embedded, gold-nanoparticle-based delivery vehicle provides efficient local, selective, and sustained release of miR-96/miR-182, markedly suppressing metastasis in a breast cancer mouse model. Combined delivery of the miRNAs with a chemotherapy drug, cisplatin, enables significant primary tumour shrinkage and metastasis prevention. Our data corroborate the role of miRNAs in metastasis, and suggest miR-96/miR-182 delivery as a potential anti-metastatic drug.

## Abstract (web summary)

MicroRNAs represent potential therapeutic targets to control metastasis progression. Here the authors show that miR-96 and miR-182 regulate invasion via Palladin and demonstrate that local delivery of miR-96 and miR-182 may serve as a potential anti-metastatic drug in breast cancer.

## Graphical abstract

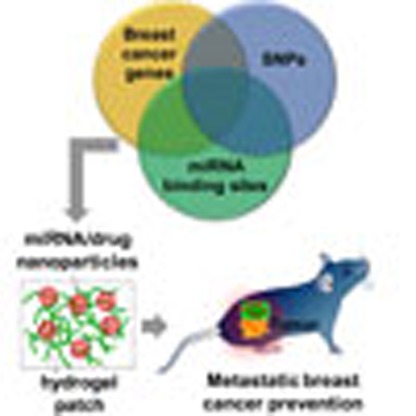

Metastases are the primary cause for mortality in breast cancer, the most common cancer in women regardless of ethnicity^1^. In fact, one in eight women are diagnosed with and develop invasive/metastatic breast cancer^2^. Metastasis is a multi-step process, comprised of a series of sequential steps, starting with epithelial-to-mesenchymal transition, local migration and invasion of cancer cells from the primary tumour to the surrounding host tissue, intravasation into blood or lymphatic vessels, dissemination via the blood or lymphatic stream, extravasation to distant organ, survival in dormancy and finally proliferation and angiogenesis within the organ^3^,^4^,^5^. Only a unique subpopulation of primary tumour cells that acquire special traits (arising from specific gene expression) that allow the cells to successfully complete all these steps will survive and produce secondary metastases^4^,^5^. Hence, each step in that process provides potential targets for metastasis prevention. However, currently, breast cancer therapy lacks effective anti-metastatic strategies^6^.

MicroRNAs (miRNAs) are non-coding small RNAs that negatively regulate gene expression. and are highly associated with tumorigenicity, invasion, and metastasis^7^. Precise sequence complementation between the seed region, comprising bases 2-8 from the 5′ end of the miRNA, and its binding-site within the 3′ untranslated region (3′-UTR) of the target mRNA is necessary to exert the downregulation effect. Recent studies show that germline sequence variants, such as single-nucleotide polymorphisms (SNPs) in miRNA-binding sites, can disrupt the downregulation by miRNAs, with a profound effect on gene expression levels and consequentially on the phenotype, including increased risk for cancer^8^,^9^,^10^. In the current study, we aim to determine the potential effect of SNPs within miRNA-binding sites on metastatic breast cancer progression and their potential use as suppression targets to prevent metastasis, representing an urgent therapeutic need^6^ (see Fig. 1 for schematic study design).

Bioinformatics analysis identified a SNP, which potentially influences breast cancer metastasis. This SNP, rs1071738, was found to be located in a target site for miR-96 and miR-182 on the 3′-UTR of the *PALLD* gene, which encodes the Palladin actin-associated protein, a documented player in breast cancer motility^11^,^12^,^13^. *In vitro* experiments revealed a functional downregulation of Palladin levels by miR-96 and miR-182, which subsequently reduces migration and invasion abilities of breast cancer cells. However, the miRNAs regulate Palladin in a SNP-genotype dependent manner, that is, Palladin regulations is disrupted by the SNP allele that abolish the binding of the miRNA to its target mRNA. Applying complimentary engineered miR-96 or miR-182 enables restoration of Palladin regulation. An *in vivo* experiment showed that the use of nanoparticles embedded in a hydrogel scaffold as a miRNA delivery vehicle enables an efficient and specific delivery of miR-96/miR-182 to breast tumours, which results in marked reduction of breast cancer metastasis. Moreover, intercalation of a chemotherapy drug, cisplatin, to the miR-conjugated nanoparticles further improves the effect, leading to significant reduction in both primary tumour growth and metastasis. Our study highlights the therapeutic potential of miRNAs, and specifically miR-96 and miR-182, and support the importance of Palladin regulation in breast cancer metastasis.

## Results

### PALLD SNP as breast cancer metastasis functional variant

**Figure 2:**
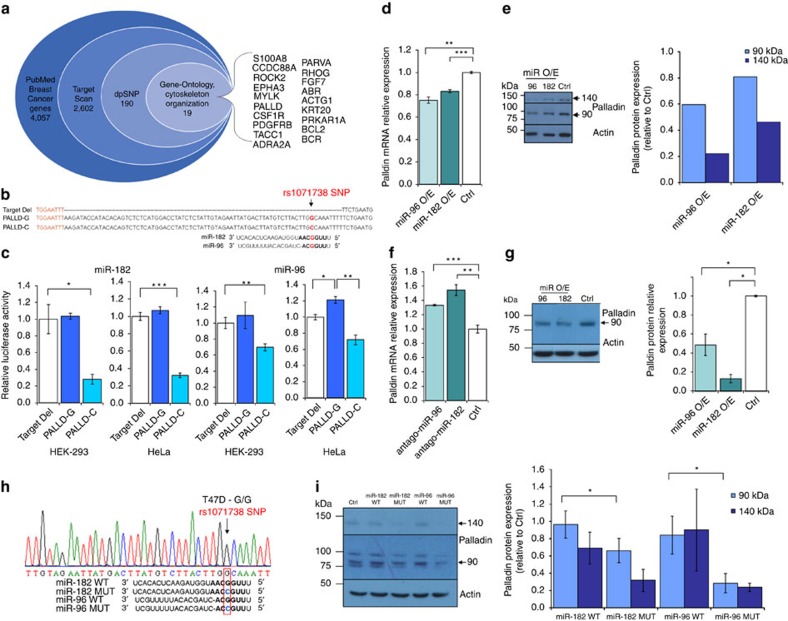
miR-96 and miR-182 regulate the Palladin metastasis promoting protein. (**a**) Stacked Venn diagram of the omic-data integration results. The outer circle represents the number of 'breast cancer genes' in PubMed (see Methods). The 2nd inner circle represents the number of genes with a conserved miRNA target site in their 3′-UTR based on TargetScan. The 3rd inner circle represents the number of genes with a known SNP in their miRNA target site based on dbSNP138common. The most inner circle represents the number of breast cancer genes with potential functional variants classified as 'cytoskeleton organization' by Gene Ontology. (**b**) Predicted binding sites for hsa-miR-96/182 on the *PALLD* 3′-UTR. rs1071738 *PALLD* SNP is marked in red. Three Luciferase constructs under regulation of the *PALLD* 3′-UTR were used for transient reporter assay experiments: negative control 3′-UTR (Target-deletion), G allele, and C allele. Below, sequences of mature hsa-miR-96 and hsa-miR-182 aligned to the target site with the ‘seed' regions marked in bold. (**c**) Luciferase activity 48 h following co-transfection with hsa-miR-96 or hsa-miR-182 in combination with either of the *PALLD* 3′-UTR constructs. (**d**) Palladin mRNA downregulation 24 h following overexpression of hsa-miR-96, hsa-miR-182 or pcDNA3 control plasmid in Hs578 cells. (**e**) Palladin protein downregulation (isoforms 3 and 4) 24 h following overexpression of hsa-miR-96, hsa-miR-182 or pcDNA3 control plasmid. (**f**) Palladin mRNA upregulation 24 h following downregulation of miR-96 or miR-182 (by antago-miRs or scrambled control) in MCF-7 cells. (**g**) Palladin protein downregulation (isoform 4) 2–3 weeks following stable overexpression of mmu-miR-96, mmu-miR-182 or scrambled control plasmid in 4T1 cells. (**h**) rs1071738 SNP genotype of T47D cell line (Sanger sequencing). Aligned below are four sequences: mature wild-type (WT) hsa-miR-96 and hsa-miR-182 (possessing a G nucleotide on the opposed position of the SNP), and mutant (MUT) miR-96 and miR-182, in which the G nucleotide was replaced by a C nucleotide. The seed regions (marked in bold) of the mutant miRNAs are fully complimentary to the binding site. (**i**) Palladin protein levels (isoform 3 and 4) 48 h following transfection of T47D cells by the indicated miRNAs (representative photos on the left). Values are presented as mean±s.e.m. (*n*=3, Student's *t*-test *P<0.05, **P<0.01, ***P<0.005).

To determine the potential effect of SNPs within miRNA-binding sites on metastatic breast cancer progression, we initially generated a cross section list of such potentially functional variants. We utilized a stepwise omic-data integration approach. In step I, we intersected a list of breast cancer genes (based on PubMed, see Methods section) with two additional data sets: TargetScan, a database of conserved miRNA target sites, and dbSNP, a database of known SNPs (Fig. 2a, Supplementary Dataset 1). In step II, we further restricted the list of genes to genes that were classified by the Gene Ontology (GO) term 'cytoskeleton organization' since one of the most critical steps in tumour progression and metastasis is the acquisition of migration and invasion capabilities^3^,^5^ by reassembly of actin-cytoskeletal structures in the cell^14^. Using this approach, we identified 20 SNPs that are located in 3′-UTR miRNA-binding sites of 19 breast cancer genes known to be involved in cytoskeleton organization. Importantly, six of these genes (>30%) were previously identified as contributors to tumour metastasis (Supplementary Table 1).

Of the potentially functional SNPs found in 'cytoskeleton organization' genes, we focused on the SNP with the highest (>43%) minor allele frequency and thus largest population effect: the rs1071738 SNP in the *PALLD* gene (Supplementary Table 1). We speculate that this type of functional variant is not under strong negative selection as its effect is exerted after the reproduction period.

The ancestral C allele of rs1071738 is the minor allele in dbSNP and in European populations, whereas the alternate G allele is much more common. However, allelic frequencies of the two alleles vary greatly between diverse populations (20–90%).

The *PALLD* gene encodes multiple isoforms of the Palladin actin-associated protein, resulting from multiple start sites and alternative splicing of the gene. Currently, the Universal Protein Database lists nine distinct Palladin isoforms^15^; however, differential expression of palladin isoforms is largely cell-type specific. The three isoforms most commonly studied are: isoform 4, with an apparent molecular mass of 90 kDa, highly expressed in vertebrates, and the most ubiquitous isoform in both embryonic and adult mouse tissues; isoform 3, 140 kDa, widely expressed and particularly abundant in the brain and in epithelial-derived cell lines; isoform 1, 200 kDa with limited expression in heart, skeletal muscle, testis and bone^16^,^17^,^18^.

Palladin isoform 4, and to some extent also Palladin isoform 3, were recently shown to be involved in the invasive behaviour of metastatic cells, specifically breast cancer cells, by increasing migration and invasive motility through several mechanisms such as linking extracellular matrix degradation to cell cytoskeleton^11^ and regulating podosome and invadopodia protrusions formation^12^,^13^. Moreover, recent studies suggest that Palladin also plays a role in gene expression regulation. Specifically, genes involved in biosynthesis and assembly of collagen as well as organization of the extracellular matrix (in cancer-associated fibroblasts)^15^, in addition to differentiation marker genes (in smooth muscle cells)^19^. Therefore, Palladin expression regulation might influence multiple cellular pathways involved in cell motility.

### miR-96/-182 regulate Palladin in a genotype-dependent manner

The *PALLD* rs1071738 SNP is located within a predicted binding site for miR-96 and miR-182. The ‘seed' regions of miR-96 and miR-182 are fully complimentary to the *PALLD* gene when the ancestral C allele is present at their binding sites, and harbor one mismatch when the alternate G allele is present (Fig. 2b). To validate the regulation of Palladin expression by miR-96 and miR-182, we initially performed a luciferase reporter assay. Significant direct downregulation of Palladin by miR-96 (∼30% reduction) and miR-182 (∼70% reduction) was observed in the presence of the complementary C allele in both HeLa and HEK-293 T cell lines (Fig. 2c). However, in the presence of the alternate G allele, Palladin regulation by miR-96/182 was abolished. Next, we examined Palladin and miRNA expression *in vitro* in two human breast cancer cell lines: MCF-7, a non-invasive breast cancer cell line, and Hs578, a highly invasive breast cancer cell line. Both cell lines are heterozygous for rs1071738 (Supplementary Fig. 1). Palladin mRNA expression was assayed by RT-PCR analysis using one set of primers which amplify Palladin isoforms 1, 3 and 4 (Supplementary Table 3). Palladin protein was detected by Western blot analysis using anti-Palladin antibody, which reacts with all three of the aforementioned isoforms (see methods). Interestingly, these cell-lines showed opposite expression profiles of Palladin and the miRNAs; in the invasive cell-line, Hs578, the expression of Palladin was relatively high and miR-96 and miR-182 low, and in the non-invasive MCF-7 cell-line, the opposite trend was observed (Supplementary Fig. 2), further supporting that downregulation of Palladin expression is controlled by miR-96/182.

In accordance with our notion, Palladin expression levels were suppressed by overexpression of miR-96 or miR-182 in Hs578 cells (mRNA and protein in Fig. 2d,e, respectively), and increased following miR-96/182 downregulation in MCF-7 cells (Fig. 2f) (for miRNA over/underexpression see Supplementary Fig. 3A and B). To further examine the mechanism of this effect *in vitro*, we utilized the highly aggressive mouse breast cancer cell line, 4T1, which is homozygous for the ancestral C allele of rs1071738 (Supplementary Fig. 1). The binding-site of miR-96 and miR-182 at the region complementary to the ‘seed' is identical and evolutionarily conserved between the human and mouse Palladin orthologs. The murine miR-96 sequence is completely identical to the human ortholog, and the miR-182 sequence differs in two nucleotides at the 3' end of the miRNA. In agreement with the human results, inducing stable expression of mouse miR-96 or miR-182 (Supplementary Fig. 3C) reduced Palladin isoform 4 levels dramatically in 4T1 cells (Fig. 2g). Palladin isoform 3 was undetectable in these cells. The prominent reduction in Palladin isoform 4 levels following overexpression of miR-96 or miR-182 may be explained by stronger binding to the C alleles at the binding site, and/or by stable expression of the miRNAs.

We hypothesized that the genotype-dependent deregulation can be ‘repaired' by applying a complimentary engineered miRNA. Using the T47D human breast cancer cell line, which is homozygous for the alternate G allele, we observed that overexpression of WT miR-96 or miR-182 did not influence Palladin isoform 4 levels, whereas overexpression of engineered miR-96 or miR-182, in which the G nucleotide on the opposed position of the SNP was replaced by a C nucleotide thereby allowing full complementation with the binding site (Fig. 2h), repaired binding and reduced Palladin isoform 4 levels (Fig. 2i). Palladin isoform 3 levels show the same trend, however the results did not reach statistical significance, possibly due to low quantification accuracy considering the very low expression levels of this isoform in T47D cells. These results clearly demonstrate a functional regulatory effect for the rs1071738 SNP, in which the ancestral C allele permits miRNA:mRNA binding and the alternate G allele disrupts binding.

### Palladin downregulation by miR-96/-182 reduces cell motility

Growing evidence highlights the role of Palladin in cell motility, however, studies investigating the role miR-96 and miR-182 take in regulating cancer cells migration and invasion processes (as well as proliferation or cell death) are in dispute. While some studies indicate an anti-invasion/migration or anti-proliferation role for miR-96 and/or miR-182 (refs 20, 21, 22, 23, 24), other studies, including studies addressing breast cancer, support the opposite^25^,^26^,^27^,^28^,^29^,^30^,^31^,^32^,^33^. Therefore, the effect of miR-96 and miR-182 on the invasive behaviour of the cells was determined.

**Figure 3:**
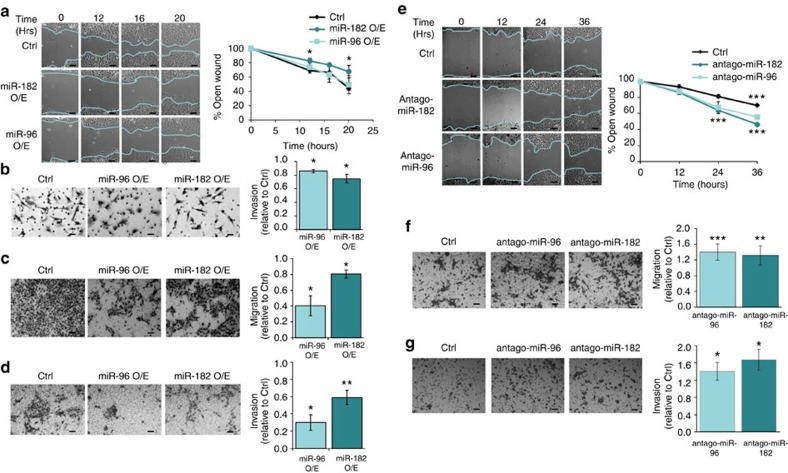
miR-96 and miR-182 reduce migration and invasion of breast cancer cells *in vitro*. Migration and invasion abilities were evaluated using wound healing, transwell migration, and Matrigel invasion assays. Hs578 cells were transfected by hsa-miR-96, hsa-miR-182 or pcDNA3 control plasmid (Ctrl). Wound healing assay results (**a**) showed migration inhibition by hsa-miR-182. Pictures were taken at the indicated time points after scratch. The graph to the right represents the width of remaining open wound calculated in relation to time 0 (*n*=3). (**b**) Overexpression of hsa-miR-96 and hsa-miR-182 reduced invasion as was demonstrated by Matrigel invasion assay. Photos of representative fields are on the left. Results were calculated as invasion rate in relation to control cells. Transwell migration assay (**c**) and Matrigel invasion assay (**d**) of 4T1 cells stably expressing mmu-miR-96 or mmu-miR-182 showed decreased migration and invasion abilities compared with 4T1 cells stably expressing scrambled sequence as control (Ctrl). Photos of representative fields are on the left. Results were calculated as migration or invasion rates in relation to control cells (*n*=3). (**e**) Wound healing assay showing increased migration following reduction of hsa-miR-182 or hsa-miR-96. MCF-7 cells were transfected by antago-miR-182, antago-miR-96 or scrambled control. Pictures were taken at the indicated time points after scratch. The graph to the right represents the width of remaining open wound calculated in relation to time 0 (*n*=4). Downregulation of mmu-miR-96 and mmu-miR-182 by antago-miR transfection enhanced migration (**f**) and invasion (**g**) of 4T1 cells. Scale bars, 50 μm. Values are presented as mean±s.e.m. (Student's *t*-test, *P<0.05, **P<0.01, ***P<0.005).

**Figure 4:**
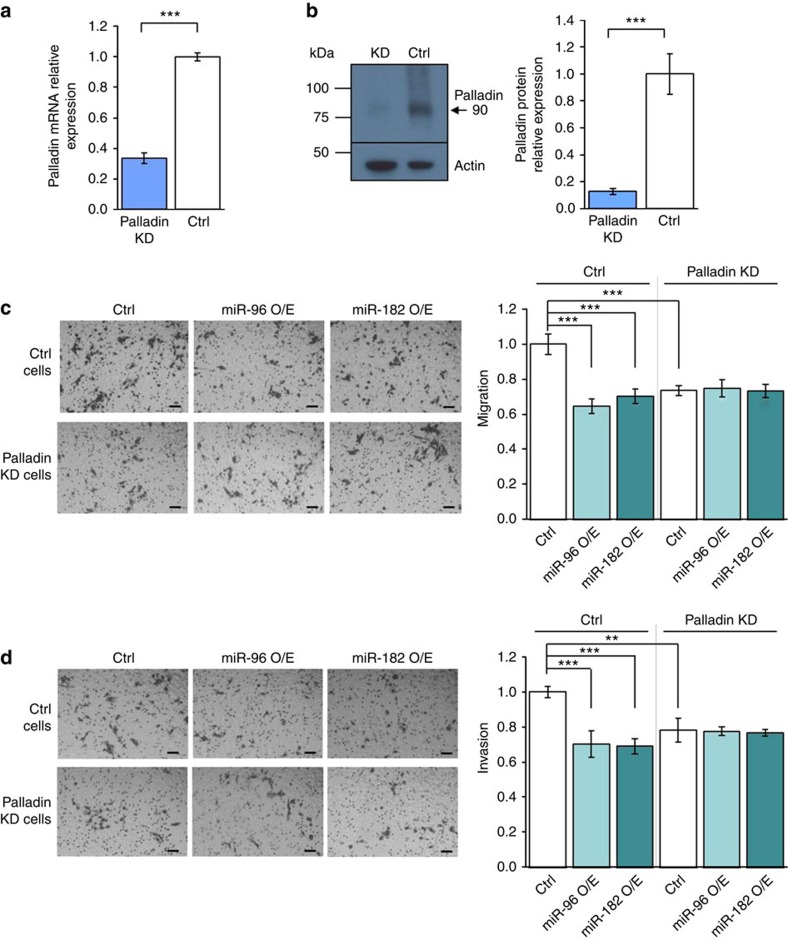
Palladin is a functional downstream target of miR-96 and miR-182. (**a**) Palladin mRNA downregulation following stable overexpression of Palladin shRNA, as assayed by qRT–PCR. RNA was extracted from 4T1 cells 3 weeks following infection with Palladin shRNA or scrambled shRNA as control. mRNA expression levels were normalized to GAPDH. (**b**) Western blot demonstrating decreased Palladin protein (isoform 4, 90 kDa) on stable overexpression of Palladin shRNA. Protein was extracted from 4T1 cells 3 weeks following infection. Protein band quantification was performed using ImageJ software and protein levels were normalized to Actin levels. Transwell migration assay (**c**) and Matrigel invasion assay (**d**) of 4T1 cells stably expressing Palladin shRNA (Palladin KD cells) or scrambled shRNA (Ctrl cells), 48 h following transfection with mmu-miR-96, mmu-miR-182 or scrambled control. Photos of representative fields are on the left (*n*⩾3). Scale bars, 50 μm. Values are presented as mean±s.em. (Student's *t*-test, **P<0.01, ***P<0.005).

Overexpression of miR-182 inhibits wound closure (by 53% after 20 h, Fig. 3a), and overexpression of both miR-96 and miR-182 inhibit invasion in transwell invasion assays (by 14% and 25%, respectively, Fig. 3b) of Hs578 human invasive breast cancer cells. Similarly, stable overexpression of both miR-96 and miR-182 in mouse 4T1 invasive breast cancer cells inhibit migration in transwell migration assays (by 59% and 19.5%, respectively, Fig. 3c) as well as invasion in transwell invasion assays (by 70% and 41%, respectively, Fig. 3d). In contrast, downregulation of both miR-96 and miR-182 in MCF-7 human non-invasive breast cancer cells enhanced wound closure (by 21% and 34%, respectively, Fig. 3e) and in 4T1 cells enhanced migration (by 40% and 30%, respectively, Fig. 3f) and invasion (by 39% and 65%, respectively Fig. 3g). As expected, short hairpin RNA (shRNA)-mediated suppression of Palladin in 4T1 cells (Fig. 4a,b) significantly reduced migration and invasion abilities (by 27% and 22%, respectively) (Fig. 4c,d). Importantly, Palladin suppression specifically blocked the inhibiting effects of miR-96 and miR-182 overexpression on migration and invasion abilities of 4T1 cells (Fig. 4c,d), thereby functionally linking the miRNAs expression, Palladin expression, and cell motility (for miRNA over/underexpression see Supplementary Fig. 3). Taken together, these data strongly suggest an anti-migration and anti-invasion role for miR-96 and miR-182 in breast cancer, mediated by Palladin downregulation.

### miR-96/-182 do not affect breast cancer cell proliferation

We evaluated the effect of miR-96 and miR-182 on proliferation rates in 4T1, MCF-7, and Hs578 breast cancer cell lines. No effect on proliferation rate was observed in 4T1 and Hs578 cell lines following overexpression of both miRNAs (Supplementary Fig. 4A and C) and in MCF-7 cells following miR-96 downregulation (Supplementary Fig. 4B). Downregulation of miR-182 in MCF-7 cells resulted in a slightly accelerated proliferation rate (∼6%; Supplementary Fig. 4B).

### *In vivo* data support miR-Palladin regulation and metastasis

Thus far, we have demonstrated the downregulation effect of miR-96/182 on Palladin expression in *in vitro* experiments. To inquire whether this effect occurs similarly *in vivo*, we examined Palladin and miR-96/182 relation in The Cancer Genome Atlas (TCGA) Breast invasive carcinoma (BRCA) cohort^34^. In agreement with the *in vitro* findings, we found that there is a negative correlation between Palladin and miR-96 or miR-182 normalized expression levels (*r*=−0.3 and *r*=−0.2, respectively; Supplementary Figs. 5 and 6). Unfortunately, we were not able to assess the effect of Palladin on metastatic state since only a few (*n*=21) samples had detectable distant organ metastasis (pathologic M1). Yet, we did observe association with lymph node metastases when adjusting for tumour size (pathologic T). Both pathologic N staging (N0–N3), and the number of lymph nodes positive by hematoxylin and eosin (H&E) were significantly increased with Palladin expression levels (*P*≤0.005, Supplementary Table 2, Supplementary Fig. 7). Importantly, it should be noted that these associations cannot be explained by miR-96 or miR-182 expression levels as no significant association was obtained between these miRNAs and lymph node metastases (*P*⩾0.1, Supplementary Table 2). Of note, the TCGA BRCA study was insufficiently powered to detect small allelic effects due to its large diversity and small sample size. Genotypes and gene expression levels were available for only 460 homogeneous samples (Caucasians), as the study-sample is comprised predominantly of Caucasians (68%), with 16% African-American, 6% Asian and 10% others (Supplementary Fig. 6), resulting in less than 30% power to detect a standardized effect-size of 0.1 (typical for SNPs) and rs1071738 allele frequency (40%). Hence, further investigation in larger breast cancer and metastatic breast cancer studies is needed to clarify the regulatory effects of the triad rs1071738, miR-96/182, and Palladin in breast cancer patients.

### miR-96 and miR-182 prevent breast cancer metastasis *in vivo*

To explore whether miR-96 and miR-182 can prevent breast cancer metastasis *in vivo*, we examined the prevalence of metastases in an orthotopic breast cancer mouse model derived from 4T1 cells that were engineered to overexpress miR-96 or miR-182 (see Supplementary Fig. 8A for experiment flow). 4T1 cells were selected because tumour growth and metastatic spread of these cells in BALB/c mice closely mimic stage IV human breast cancer. Real-time PCR analysis validated increased miR-96 and miR-182 levels (Supplementary Fig. 8F) and decreased Palladin levels (Supplementary Fig. 8G–H) in removed primary tumours stably expressing miR-96 or miR-182, respectively, compared with tumours stably expressing control miRNA. A profound decrease in the appearance of lung metastatic nodules was found in tumours stably expressing miR-96 or miR-182 compared with tumours stably expressing the scrambled control (Supplementary Fig. 8I–L), with no effect on primary tumour characteristics (Supplementary Fig. 8B–E).

### miR-96 and miR-182 delivery suppress metastasis *in vivo*

**Figure 5:**
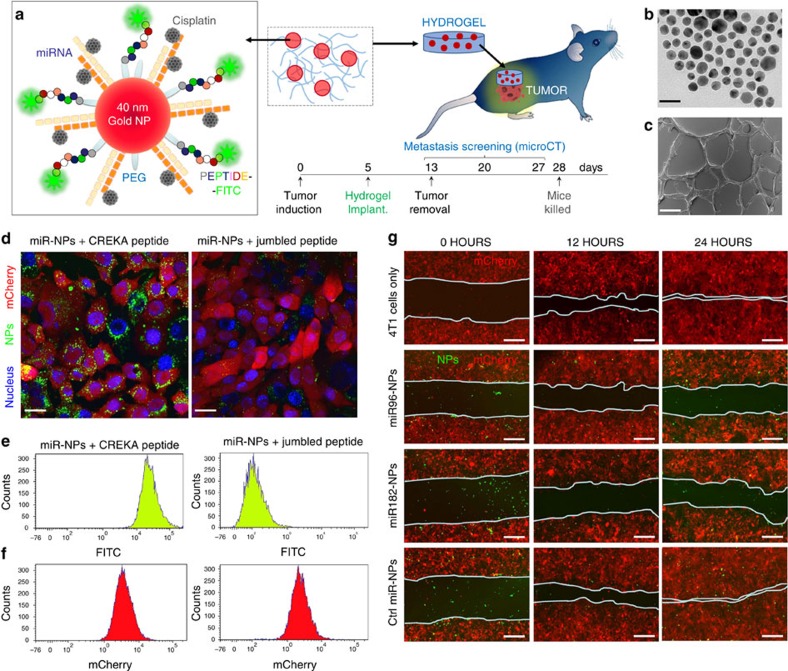
Nanoparticle-based strategy to deliver miR-96 and miR-182. (**a**) Dextran-dendrimer hydrogels embedded with targeted nanoparticles. Targeted miR nanoparticles consist of gold nanoparticles (AuNPs, ≈40 nm diameter) functionalized with thiolated-PEG-COOH conjugated to 4T1-targeting peptide (CREKA) labelled with FITC, thiolated miRs and cisplatin intercalated on the miR strands. (**b**) High-resolution TEM images of the gold nanoparticles. Scale bar, 50 nm. (**c**) High-resolution SEM images of the dextran–dendrimer hydrogel scaffolds. Scale bar, 100 μm. (**d**) Confocal microscopy and (**e,f**) flow cytometry of the cellular uptake in 4T1 cells of miRNA-nanoparticles functionalized with the CREKA peptide in comparison with a jumbled peptide. Nanoparticles in green (FITC) and mCherry-expressing 4T1 cells (in red). Nuclei (in blue) were stained with DAPI. Scale bars, 25 μm. (**g**) Wound healing assay performed in 4T1 cell lines using the gold NP carriers for miR-96, miR-182 or Ctrl miR at 0, 12 and 24 h after incubation. Nanoparticles in green (FITC) and mCherry-expressing 4T1cells (in red). Scale bars, 150 μm.

After demonstrating the functional role of miR-96 or miR-182 in metastasis prevention, in a first step towards translation, we sought to test the therapeutic anti-metastatic potential of Palladin modulation by administrating miR-96 or miR-182 externally, carried on a suitable vehicle. It is well documented that Palladin isoform 4 is abundantly expressed in all types of smooth muscle cells (vascular and visceral)^18^,^19^,^35^, hence it is reasonable to assume that a systemic approach targeting Palladin will have disastrous side effects and local administration is needed. We hypothesized that local and efficacious delivery of miRNA mimics can be achieved by coating 4T1 breast tumours with an adhesive hydrogel scaffold/patch (made of oxidized dextran and PAMAM G5 dendrimer) embedded with nanoparticles carrying the miRNAs of interest (Fig. 5a). The cationic surface of the dendritic system provides a facile platform for the incorporation of miRNA nanoparticles forming electrostatic interactions between the positively charged terminal amines from PAMAM dendrimers and the negatively charged nanoparticles. We have previously shown the enhancement of nanoparticles stability *in vivo* when embedded in a hydrogel for sustained gene and drug delivery applications^36^,^37^,^38^,^39^.

In addition, to test the efficacy of miRNA treatment for metastasis prevention as an add-on to conventional chemotherapy, we further leveraged the local miRNA delivery scaffold that coats the primary tumour to simultaneously deliver a chemotherapy drug, cisplatin, and compared the outcome following administration of nanoparticles with only miRNAs to that of nanoparticles with both miRNA and cisplatin. Cisplatin is an alkylating agent classified as an anti-neoplastic drug that has been extensively used in treatment of advanced breast cancer, especially in metastatic breast cancer and, more recently, in triple-negative breast cancer^40^,^41^,^42^,^43^. Moreover, its chemical structure enables its intercalation to the miRNAs thus providing sustained rather than bolus release over time.

The nanoparticles used in this study consist of a ≈40 nm gold core that was decorated with thiolated miRNAs and a targeting peptide. Engineered miR-96 and miR-182 oligos were bound to the gold surface by the strong interaction of thiol groups (at the 5′ end of the miRNA oligos) and the gold core, forming a quasi-covalent interaction. Thiolated-PEG-COOH enables conjugation to the 4T1-targeting peptide (CREKA), which was labelled with fluorescein isothiocyanate (FITC; Fig. 5a). The pentapeptide CREKA (Cys-Arg-Glu-Lys-Ala) is a tumour-homing pentapeptide that specifically ‘homes' to fibrin-fibronectin complexes abundantly expressed in tumour microenvironments and specifically binds to 4T1 breast cancer cells^44^. We thus expected that, with local delivery, the peptide would improve cellular uptake.

Implantable dendrimer-dextran hydrogels were doped with the drug/miRNA-gold nanoparticles to afford local drug/miRNA delivery to shrink the primary tumour and prevent metastasis. Quantification (Supplementary Fig. 9) and characterization assays (Fig. 5b,c, Supplementary Figs 10 and 11) were conducted for the functionalized nanoparticles. Absorption band of the resulting nanoparticles appeared between ∼530 to ∼550 nm due to the surface plasmon resonance (SPR) of the nanoparticles (Supplementary Fig. 11A). The spatial arrangement of nanoparticle can be inferred by measuring the shift of a characteristic SPR band dependent on size, shape, aggregation state, and medium polarity (Supplementary Fig. 11B). The SPR of the nanoparticles shown exhibited a red shift from 528 to 546 nm: a 5 nm shift for poly(ethylene glycol) (PEG) binding, a 5 nm shift for CREKA peptide binding and an 8 nm shift for miRNA binding indicating successful binding of the several groups. Dynamic light scattering measurements with diameter distribution histograms (Supplementary Fig. 11C) and Zeta Potential (Supplementary Fig. 11D) of the resulting nanoparticles also confirm the successful functionalization of PEG, peptide and miRNA. The quantification of all biomolecules (that is, PEG, peptide and miRNAs) functionalized on the surface of gold nanoparticles can be found in Supplementary Fig. 12. With a remarkable loading capacity (miRNA:nanoparticle ratio around 250:1) these nanoparticles may represent an efficient therapeutic route for miRNA delivery.

Confocal microscopy (Fig. 5d) and flow cytometry (Fig. 5e,f) were used to evaluate the cellular uptake of cells incubated with miRNA nanoparticles functionalized with the CREKA peptide in comparison with a jumbled peptide, showing that the nanoparticles carrying the targeting peptide CREKA have two orders of magnitude higher cellular uptake than the ones carrying a jumbled peptide.

To confirm that the miRNAs maintain their function when conjugated to the gold nanoparticles, we evaluated 4T1 cell motility by performing a wound healing assay following treatment with the miRNAs carried by the nanoparticles *in vitro* (Fig. 5g). The results show that cells treated with nanoparticles carrying a control miRNA were able to close the wound almost completely within 24 h. Treatment with gold nanoparticles carrying the miR-96/-182 dramatically inhibits wound closure in 4T1 compared with treatment with Control-miR nanoparticles. Cells treated with nanoparticles carrying either miR-96 or miR-182 were not able to close the wound completely. These findings indicate that the delivery of either miR-96 or miR-182 abolishes the motility of 4T1 cells. We confirmed the enhancement of miR-96 and miR-182 expression level following treatment with the gold nanoparticles carrying the miR-96/-182 or control miR using quantitative PCR (Supplementary Fig. 13). As expected, the Palladin levels were downregulated after treatment with nanoparticles-miR-96 or nanoparticles-miR-182. These results demonstrate that utilizing gold nanoparticles, enables delivery of functional miRNAs, leading to decreased cell motility *in vitro*.

We next assessed the *in vivo* pharmacokinetic and therapeutic profile of the miRNA-nanoparticles doped hydrogel scaffold in an orthotopic metastatic breast cancer mouse model. Tumours in the mammary fat pad were induced in BALB/c female mice (BALB/cAnNCrl) by injection of 4T1 cells stably expressing mCherry. Hydrogel scaffolds loaded with the miRNA-nanoparticles were implanted adjacent to the tumour in the mammary fat pad when tumours reached the desired volume (∼100 mm^3^, about 5 days after tumour induction). Seven days after hydrogel implantation the primary tumours were removed and the presence of metastases in the lungs was evaluated by micro-computed tomography (micro-CT) for 14 additional days. Then, mice were sacrificed and organs collected and screened for the presence of macro-metastases.

**Figure 6:**
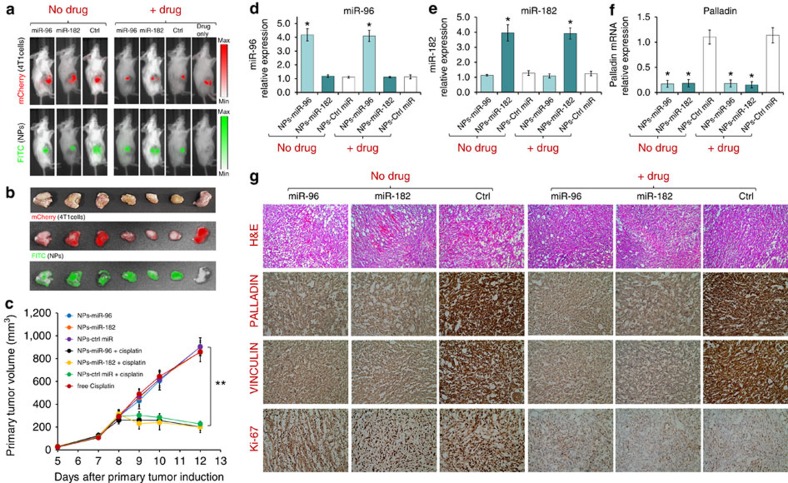
*In vivo* delivery of miR-96 and miR-182 via targeted nanoparticles targets the primary tumour in an orthotopic breast cancer model. (**a**) Live imaging of BALB/c mice with 4T1 (expressing mCherry) breast tumours induced in the mammary fat pad and implanted with hydrogels embedded with targeted gold NPs carrying miR-96, miR-182 or scrambled (Ctrl) miRNAs with and without cisplatin. (**b**) *Ex vivo* fluorescent images of breast tumours to evaluate mCherry expression and NPs (FITC) accumulation. (**c**) Primary tumour volume following treatment. Quantitative PCR (qPCR) determination of (**d**) miR-96, (**e**) miR-182, and (**f**) Palladin mRNA expression levels in mice treated with hydrogels embedded with targeted gold NPs carrying miR-96, miR-182 or scrambled (Ctrl) miRNAs with and without cisplatin. (**g**) H&E and immuno-histochemistry for Palladin, Vinculin and Ki-67 of resected tumours from treated mice. All values are presented as mean±s.e.m. (*n*=5, Student's *t*-test, *P<0.05, **P<0.01, ***P<0.005).

Primary tumour progression was measured by mCherry expression (emission at 620 nm) while each miRNA-nanoparticles release was tracked fluorescently via live imaging system 7 days post-hydrogel implantation. Live imaging of mice and *ex vivo* fluorescent images of breast tumours revealed that FITC-labelled nanoparticles were able to accumulate similarly in tumours from all treated groups (Fig. 6a,b). This confirms the capacity of this platform to provide an efficient *in vivo* miRNA mimic delivery. No signs of inflammation at the surgical site, nor changes in body weight, were observed before or after breast tumour induction or hydrogel implantation (Supplementary Fig. 14), suggesting that hydrogels and nanoparticles are biocompatible with no associated toxicity or side effects.

The hydrogel scaffolds doped with nanoparticles carrying both miRNAs and the chemotherapeutic drug (cisplatin), compared with only miRNAs, showed lower mCherry expression in tumours, as was demonstrated by live mouse imaging (Fig. 6a) and *ex vivo* fluorescent images of breast tumours (Fig. 6b). This implicates efficient inhibition of the primary tumour progression due to the effect of cisplatin release. More than 50% tumour size reduction was observed for cisplatin-containing nanoparticles 7 days after hydrogel disk implantation (Fig. 6c), with a two-fold reduction in tumour weight (Supplementary Fig. 15).

To evaluate the expression of miR-96 and miR-182 and their effect on Palladin expression, gene expression analysis of resected tumours was conducted. Indeed, real-time PCR results showed a approximately fourfold increase in both miR-96 and miR-182 following treatment with hydrogels embedded with targeted nanoparticles carrying miR-96 or miR-182 with and without cisplatin, compared with the control miRNA (Fig. 6d,e). Interestingly, tumours showed inverse expression levels of Palladin and the miRNAs: Palladin mRNA expression was high only in groups treated with hydrogels embedded with targeted nanoparticles carrying control miRNA and exhibited a approximately fivefold decrease following treatment with miR-96 or miR-182, which was not weakened by the intercalation of cisplatin to the miRNA-nanoparticles (Fig. 6f). Hence, Palladin mRNA expression levels were suppressed by delivery of miR-96 or miR-182.

Extensive reduction of vascularization in the cisplatin treated groups was evidenced by H&E staining of breast tumour sections, when compared with miRNA delivery only (Fig. 6g). Immunohistochemical (IHC) analysis corroborated that the expression of Palladin and Vinculin (which also possesses a conserved binding site for miR-96 and miR-182 on its 3′-UTR, according to TargetScan) was extensively reduced with overexpression of miR-96 or miR-182, validating the quantitative PCR results (Fig. 6d-f). In fact, both Palladin and Vinculin are cytoskeletal proteins associated with cell–cell and cell–matrix junctions, required for organizing the actin cytoskeleton. Therefore, as the overexpression of miR-96 and miR-182 downregulates Palladin levels, a reduction in migration and invasion abilities occurs, as demonstrated by the *in vitro* assays depicted in Figs 2. Further, IHC analysis of Ki-67, a cellular marker associated with cell proliferation, revealed that a decrease in this protein is mainly observed in groups treated with cisplatin, independent from the specific miRNA treatment (Fig. 6g). This reveals that the treatment of the primary tumour with a chemotherapeutic drug reduces cancer cell proliferation, with a concomitant reduction in tumour size.

**Figure 7:**
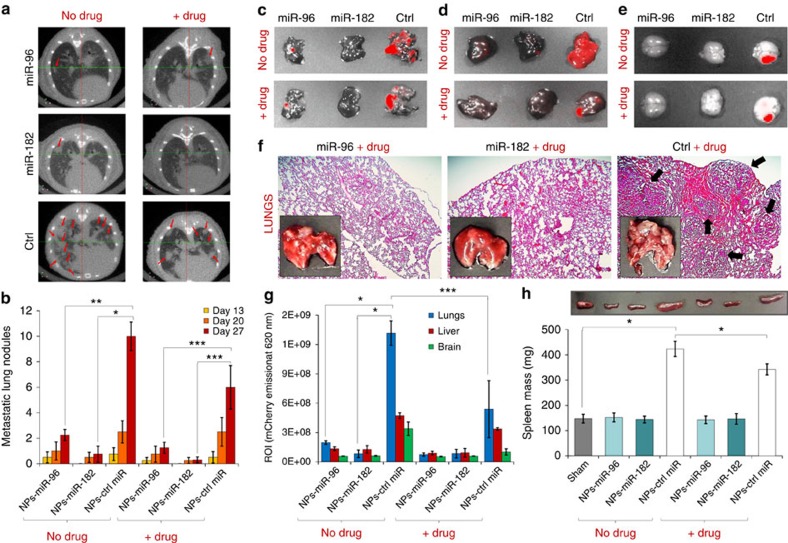
*In vivo* delivery of miR-96 and miR-182 via targeted nanoparticles prevents metastasis. (**a**) micro-CT images of the lungs in mice treated with gold NPs carrying miR-96, miR-182 or scrambled (Ctrl) miRNAs with and without cisplatin. Arrows show the presence of macro-metastasis. (**b**) Quantification of metastatic lung nodules in treated mice for days 13, 20 and 27 after primary tumour induction. Ex-vivo fluorescent images of lungs (**c**), liver (**d**) and brain (**e**) depicting mCherry emission in treated mice. (**f**) H&E stains of the resected tumours from mice treated with hydrogels embedded with targeted NPs carrying miR-182, miR-96 or scrambled (Ctrl) miRNAs with cisplatin (*n*=5 per group). Arrows depict tumour clones. (**g**) Quantification of mCherry emission at 620 nm from lungs, liver and brain. (**h**) Mass range of spleens from treated mice, when compared with non-treated (Sham). All values are presented as mean±s.e.m. (*n*=5, Student's *t*-test, *P<0.05, **P<0.01, ***P<0.005).

Knowing the potential invasive and metastatic profile of 4T1 cells, especially to lungs but also liver and brain^45^, we next evaluated the effect of enhancing miR-96 and miR-182 expression through local miRNA-mimic delivery on the establishment of *in vivo* metastasis. Metastases formation was evaluated 13 days post-tumour resection using micro-CT of the lungs (Fig. 7a). The quantification of metastatic lung nodules in treated mice for days 13, 20 and 27 after primary tumour induction (which corresponds to 0, 7 and 14 days post tumour resection) revealed that the number of nodules increased with time only for groups treated with nanoparticles carrying the control miRNA, and more pronounced in the groups with no drug delivery (Fig. 7b). *Ex vivo* fluorescent images of lungs (Fig. 7c), liver (Fig. 7d) and brain (Fig. 7e) depicting mCherry emission in treated mice, revealed the presence of 4T1 cells (migrated from the mammary primary tumour) mainly in groups treated with nanoparticles carrying the control miRNA. H&E stains of the resected tumours revealed the presence of macro-metastasis in lungs only for mice treated with hydrogels embedded with targeted nanoparticles carrying scrambled (Control) miRNAs (Fig. 7f). In fact, the mCherry emission at 620 nm is higher in lungs, but also present in liver and brain mainly for mice treated with hydrogels embedded with targeted nanoparticles carrying scrambled (Control) miRNAs (Fig. 7g). No metastases were detected in any other major organs (Supplementary Fig. 16).

Interestingly, only groups treated with nanoparticles carrying the control miRNA, with or without the drug delivery, displayed enlarged (approximately fourfold) spleens (that is, splenomegaly; Fig. 7h). Actually, the mammary tumour induced with 4T1 cells is known for presenting splenomegaly^46^,^47^, which is associated with a severe state of diseases, especially liver infections and some cancer types. In our studies, we demonstrated that the 4T1 tumour induces a leukemoid reaction with splenomegaly following orthotopic transplant into the mammary fat pads of female BALB/c mice.

## Discussion

In this study we initially identified a common functional variant, rs1071738, at the miR-96 and miR-182 target site on the *PALLD* 3′-UTR, that we thought may potentially influence breast cancer metastasis. Subsequently, we showed for the first time that Palladin expression is specifically regulated by miR-96 and miR-182, and demonstrated that the *PALLD* SNP is indeed a functional variant, in which the alternate allele abolishes miRNA:mRNA binding leading to uncontrolled regulation of Palladin expression. Importantly, we were able to restore this abnormal regulation by the administration of engineered, fully complimentary, miRNAs. *In vivo* delivery of miR-96 or miR-182 carried on nanoparticles which are embedded in a hydrogel scaffold patch and implanted adjacent to the tumour, allowed local and sustained release of the miRNAs and efficiently blocked breast cancer from spreading to lungs, liver and brain in an orthotopic mouse model, with no systemic side effects. Moreover, by intercalating cisplatin, a chemotherapy drug, within the miR-conjugated nanoparticles, we were able to achieve a local and specific delivery of the chemotherapy drug to the tumour, with a sustained release over time (as opposed to the intravenous standard of care). This delivery of the combined therapy (cisplatin and each of the miRNAs) further improved preclinical outcomes, leading to substantial reduction in both primary tumour growth and metastasis, thereby plausibly introducing our miRNA therapy as an add-on to conventional chemotherapy.

The statistically significant inverse relationship between Palladin and miR-96/182 expression, as well as the positive relationship between Palladin expression and lymph node metastases in invasive breast carcinoma cells of the TCGA study samples, further support our *in vitro* and *in vivo* findings, and highlight their relevancy. The TCGA BRCA study size was insufficiently powered to detect small allelic effects (<30% for rs1071738 minor allele frequency). Furthermore, genetic association in tumours pose additional challenges and confounding effects, such as copy number variation and within and between heterogeneity. Hence, determining the effect of rs1071738 SNP on miR-96/182 regulation of Palladin expression and breast cancer metastasis, warrants investigation of large primary and metastatic breast cancer cohorts, to promote the development of a more effective, individualized, anti-metastatic therapy.

The therapeutic anti-metastatic potential of Palladin modulation by administrating miR-96 or miR-182 may extend to other types of cancer^47^,^48^. In renal cell carcinoma, for example, elevated Palladin levels in the stroma were correlated with poor clinical outcome^49^. Another study^15^ showed that Palladin is highly expressed in the stroma of multiple tumour types including lung, stomach, colon and pancreas cancer. Additional studies have indicated a role for Palladin in pancreatic cancer^48^,^50^; abnormal and overexpression of Palladin was found in familial and sporadic pancreatic cancer tissues, leading to actin-cytoskeletal changes and increased invasive and migratory abilities^50^.

As master regulators of gene expression, each miRNA potentially regulates multiple target genes. In many cellular pathways, the effect of a single miRNA-mRNA interaction might be minor. However, the combined effect of the miRNA on several target genes which share the same biological pathway might be significant and therefore result in measurable phenotypic consequences^28^. Previous findings suggested several target genes for miR-96 or miR-182, which influence cancer cell motility: miR-96 was reported to directly regulate KRAS^20^, Ezrin^21^, NUAK1 (ref. 22), RECK^25^,^27^, FOXO3a^30^, MTSS1 (ref. 31), ephrinA5 (ref. 29), Foxf2 (ref. 23) and FOXO1 (ref. 32); miR-182 was also shown to target MTSS1 (refs 28, 31), ephrinA5 (ref. 29), Foxf2 (ref. 23), FOXO3 (ref. 33) and FOXO1 (ref. 32) as well as MITF^33^, PAI1, TIMP1 and RSU1 (ref. 28). Like Palladin, three of these proteins (Ezrin, ephrinA5 and MTSS1) were also annotated by Gene Ontology as ‘actin cytoskeleton organization' genes. However, the effect of miR-96 and miR-182 on cell motility and metastasis via regulating validated targets appears to differ between cancer types and even between cancer cell lines. Our current study delineates the network of genes regulated by miR-96/182 in breast cancer by pinpointing Palladin as an important target of miR-96 and miR-182, influencing cell motility and metastasis in breast cancer cells.

Currently, screening programs are the standard of care resulting in high incidence of invasive breast cancer diagnosis at an early stage. Our proposed treatment could potentially be administered to breast cancer patients shortly after malignant disease is confirmed, before surgical intervention, and could assist in reducing distal metastasis. In addition, the treatment can be applied locally as a washout procedure following resection to eliminate remaining malignant cells that may cause tumour recurrence and metastasis.

Taken together, this study introduces the therapeutic potential of miRNAs in breast cancer metastasis prevention. A smart delivery vehicle was developed to enable efficient, local and sustained release of miRNAs, as well as combined therapy of miRNAs with a chemotherapy drug. This novel combined therapy could further improve clinical outcome by significantly reducing primary tumour mass, as well as reducing—or even preventing—metastasis.

## Methods

### Bioinformatics

To identify potential functional variants for breast cancer progression we utilized a stepwise omic-data integration approach. In step 1, we searched PubMed with the term ‘breast cancer' and gene name and gene symbol of all HUGO Gene Nomenclature Committee (HGNC)^51^ approved genes. Out of the 19,064 HGNC genes a total of 7,608 had at least one publication with ‘breast cancer' between years 2000 and 2013 (current year at the time). We considered genes with ⩾4 publications (Q_50_=4) as breast cancer genes (*n*=4,057). Setting the cutoff to the median minimizes weak associations with breast cancer (false positives), yet is sufficiently inclusive (4,057 of 19,064 HGNC genes, ∼20%). In step 2, we restricted the breast cancer genes to genes with conserved miRNA target sites in their 3′-UTR based on TargetScan (11,161 genes with conserved miRNA target in db)^52^, resulting in 2,602 genes, and in step 3, we restricted the genes to those with a common (⩾1%) SNP located in the miRNA target sites based on the dbSNP138common database (12,896, 132 SNPs in db), resulting in a total of 190 genes and 212 variants. The R package 'RISmed' was used to retrieve information from PubMed^53^. The RefSeq, and dbSNP138common databases were downloaded from the UCSC genome annotation database for the Feb. 2009 assembly of the human genome (hg19), and overlaps between genomic intervals were calculated by the R package ‘GenomicFeatures'^54^. The final gene list was annotated by Gene Ontology biological process classifications using the R packages ‘clusterProfiler'^55^. Variants from genes classified by the term ‘cytoskeleton organization' (*n*=19) were considered as potential functional variants for breast cancer progression to metastasis.

### Cell culture

HEK-293 T, HeLa, Hs578, MCF-7 and T47D cell lines were cultured in DMEM supplemented with 10% FBS (GIBCO) and 1% L-glutamine, 100 units per ml penicillin, and 100 units ml^−1^ streptomycin (Biological Industries, Kibbutz Beit Haemek, Israel). The 4T1 cell line was cultured in RPMI (GIBCO) supplemented with 10% FBS (GIBCO), 1% L-glutamine, 1 mM sodium pyruvate, 100 units per ml penicillin, 100 units per ml streptomycin, 10 mM HEPES buffer (Biological Industries, Kibbutz Beit Haemek, Israel) and 2.5 g l^−1^ D-Glucose (Sigma). Cells were incubated at 37 °C in 5% CO_2_ atmosphere. Hs578, MCF-7 and T47D cell lines were received from Prof. Ilan Tsarfaty (Tel-Aviv University). The 4T1 cell-line was received from Prof. Ronit Satchi-Fainaro (Tel-Aviv University). HeLa and HEK-293 T cell-lines were purchased from the American Type Culture Collection (ATCC). STR profiling (DNA Diagnostics Centre, UK) and mycoplasma testing (Biological Industries) were conducted for each cell line before use.

### Constructs

For luciferase reporter assays, fragments of the *PALLD* 3′-UTR spanning the miRNA-96/182 binding sites were amplified from human genomic DNA and cloned downstream to the Renilla Luciferase Reporter of the psiCHECK-2 plasmid (Promega) that contain a Firefly Luciferase Reporter (used as control) under a different promoter. Three Luciferase constructs under regulation of the *PALLD* 3′-UTR were prepared (Fig. 2b): 3′-UTR fragment possessing a G allele in the rs1071738 *PALLD* SNP position; 3′-UTR fragment possessing a C allele in the rs1071738 *PALLD* SNP position; negative control 3′-UTR in which the miRNA-96/182-binding site was deleted by restriction enzymes.

For miRNA overexpression, Pre-miRNAs (hsa-miR-96, hsa-miR-182) were cloned into the miRNA expression vector miRVec that was provided by Prof. R. Agami^56^. Vectors expressing mutant hsa-miR-96/182 were generated by mutating the miRVec plasmids expressing WT hsa-miR-96/182 using QuikChange Lightning site-directed mutagenesis kit (Agilent Technologies).

For transient and stable overexpression of mouse miRNA-96/182, Pre-miRNAs (mmu-miR-96/182) were amplified from DNA of 4T1 cells and cloned downstream of the CMV promoter of the CD515B-1_pCDHCMV-MCS-EF1- Hygro Lentivirus Expression Vector (Tarom).

### Transfection

HeLa, HEK-293 T, MCF-7, Hs578, T47D and 4T1 cells were transfected when cells were 50–75% confluent. RNA sequences or DNA plasmids were transfected together with transfection reagent in Opti-MEM serum (Biological Industries). HEK-293 T cells were transfected using TransIT-LT1 Transfection Reagent (Mirus) and all other cells were transfected with Lipofectamin 2000 transfection reagent (Invitrogen). For miRNA overexpression studies, 0.5 μg of miRVec plasmid (for human cell lines) or CD515-B plasmid (for murine 4T1 cell line) were transfected. For miRNA inhibition studies, 30 pmole antagomiRs (Ambion) or scrambled control RNA sequence were transfected. GFP was transfected as a control and its detection was confirmed 24 h following transfection. Cells were harvested for RNA extraction, protein extraction, or lysate preparation 24–48 h following transfection.

### Dual luciferase reporter assay

HEK-293 T or HeLa cells were seeded in 24 wells plate. At ∼60% confluence, cells were co-transfected with the 5 ng psiCHECK-2 containing the desired 3′-UTR and 485 ng miRVec containing the desired pre-miRNA. Forty-eight hours following transfection, lysates were extracted and Firefly and Renilla Luciferase activities were measured using the Dual-Luciferase Reporter Assay System kit (Promega) and a Veritas microplate luminometer.

### miRNA/mRNA expression levels determination

Total RNA from cell lines was extracted using TRIzol reagent (Invitrogen, Life Technologies). RNA from primary tumour samples was extracted from frozen tissues by homogenization by TissueLyser LT (Qiagen) in TRIzol reagent according to the manufacturer's instructions (Invitrogen, Life Technologies). RNA quality was measured using NanoDrop (Thermo Scientific). cDNA for miRNA and mRNA was synthesized from total RNA. Reverse transcription reaction for mRNA was conducted with random primer and SuperScript III reverse transcriptase (Invitrogen). Reverse transcription for specific miRNAs was performed with TaqMan miRNA Assays (Applied Biosystems; ABI). Single miRNA/mRNA expression was tested similarly using TaqMan Universal PCR Master Mix (No AmpErase UNG; Applied Biosystems) or SYBR green PCR master mix (Applied Biosystems), respectively, using StepOnePlus real-time PCR system (Applied Biosystems). Specific primer pairs for mRNA expression detection were ordered from Sigma (Supplementary Table 3). Palladin mRNA quantification was performed by primers that amplify isoforms 1, 3 and 4 (Supplementary Table 3). Expression values were calculated based on the comparative threshold cycle (Ct) method. miRNA levels were normalized to U6 snRNA and mRNA expression levels were normalized to human GAPDH or mouse Actin.

### Western blot analysis

Cells were homogenized with lysis buffer and debris was removed by centrifugation. Protein concentrations were determined using the Bio-Rad protein assay (Bio-Rad Laboratories). Lysates were resolved by SDS–PAGE through 4–12% gels (GeBaGel) and transferred by electroporation to nitrocellulose membrane. Membranes were blocked for 1 hour in TBST buffer containing 5% milk, blotted with anti-Palladin (ProteinTech, cat# 10853-1-AP) or anti-Actin (Millipore, clone C4, cat# MAB1501) primary antibodies for 18 h, followed by secondary antibody linked to horseradish peroxidase. The anti-Palladin antibody was generated against the C-terminal 385 amino acids of palladin, recognizes most Palladin isoforms except isoform 6. Immunoreactive bands were detected with enhanced chemiluminescence reagent (Thermo Scientific). Band quantification was performed using ImageJ software (National Institutes of Health) and protein levels were normalized to Actin levels.

### Generation of miR-182/96 stably expressing cells

CD515B-1 Lentivectors expressing mmu-miR-96, mmu-miR-182 or a scrambled sequence were prepared as described. Packaging was done in HEK-293 T cells with pPACKH1 Lentiviral vector packaging (SBI). Forty-eight hours following HEK-293 transfection, virions containing supernatants were collected. 1 M Hepes (Biological industries) was added at a 1:20 ratio, supernatants were filtered, supplemented with 5 μl ml^−1^ polybrene (Sigma) and stored at −80 °C for further use. 4T1-mCherry cells at 50% confluence were infected with the lentiviruses in a six-well plate. Selection was done under the pressure of 200 μg ml^−1^ Hygromycin (Megapharm).

### Generation of palladin knock-down cells

Palladin knock-down was performed using shRNA sequences (Dharmacon) based on the RNAi Consortium (TRC) by the Broad institute. The target sequence on Palladin coding region was: 5′- GCTAACCTATGAGGAAAGAAT -3′. Scrambled shRNA sequence was used as control. The lentiviral vector pLKO.1 was used for shRNA expression. Packaging was done in HEK-293 T cells with ViraPower Lentiviral packaging mix (Invitrogen). Forty-eight hours following HEK-293 transfection, virions containing supernatants were collected and stored at −80 °C. Before use, supernatants were filtered and supplemented with 5 μl ml^−1^ polybrene (Sigma). 4T1 cells at 40% confluence were infected with the lentiviruses in a six-well plate and selection was done under the pressure of 2 μg/ml Puromycin (A.G. Scientific).

### Wound healing assay

Hs578, MCF-7 or 4T1 (stably expressing mCherry) cells were cultured in complete growth media until ∼90% confluence. Cells were conditioned for 5–8 h in DMEM media (Hs578 and MCF-7) or RPMI media (4T1) supplemented with 0.1% FBS, and then adherent cell monolayers were scratched with a 10 μl pipette tip and cultured in complete medium. Cells were allowed to close the wound for 20 h (Hs578), 24 h (4T1) and 36 h (MCF-7), and were observed under phase-contrast microscopy. 4T1 cells were also observed under fluorescent microscopy (using Nikon Eclipse Ti Epi-fluorescence microscope). The percentage of wound closure was assessed in relation to time 0 by ImageJ software (National Institutes of Health).

### Transwell migration and invasion assays

Migration and invasion abilities of breast cancer cells were assessed based on the area covered with cells invading through either transwell inserts (Costar) for migration assays or Matrigel-coated invasion chambers (BD Biosciences), both possessing 8 μm pores. Forty-eight hours following transfection, and 16 h following starvation in cell culture media supplemented with only 0.1% FBS, Hs578 and 4T1 cells were trypsinized and seeded at 0.5 × 10^5^ and 1 × 10^5^ cells per well, respectively, into Transwell chambers (for migration or invasion assays). 4T1 cells stably expressing miRNAs were conditioned overnight in their growth media, supplemented with only 0.1% FBS, and then trypsinized and seeded at 1 × 10^5^ cells per well into transwell chambers. The lower chamber contained complete media as chemoattractant. Cells were allowed to migrate/invade for 20–24 h, and then wells were fixed with cold Methanol, washed with PBS and stained by Hemacolor for microscopy (Merck). The non-migrating/invading cells on the upper surface of the insert were removed. The cells that had migrated to the basal side of the membrane were visualized with a Nikon Eclipse Ti microscope at 200 × magnification. Pictures of 5–10 random fields from three replicate wells were obtained and the percentage of coveredarea was assessed using ImageJ software.

### Cell proliferation assay

Proliferation rates for 4T1 and MCF-7 cells were measured using the FITC BrdU Flow Kit (BD Biosciences) according to the manufacturer's instructions. Twenty-four to forty-eight hours following transfection, cells were incubated with Bromodeoxyuridine (BrdU) for 30 min. BrdU and DAPI expressions were detected by the Gallios FACS instrument and determined by Flowing software 2. Proliferation rate for Hs578 cells was measured 48 h following transfection using ViaLight Plus Cell proliferation and cytotoxicity assay (Lonza), according to the manufacturer's instructions.

### Statistical analysis of TCGA BRCA data

The RNA and miRNA-sequencing and clinical data of BRCA study samples were obtained from The Cancer Genome Atlas (TCGA) Data portal (Level 3, open access)^34^, and available for 1,203 (mRNA) and 1,176 (miRNA) women, after excluding 12 males. Gene-level transcription estimates in RSEM normalized count were retrieved, and utilized in the statistical analyses. Correlations between normalized transcript counts were measured using the Pearson's method. We used ANOVA to test the association between Palladin expression and lymph node metastasis while controlling for other staging factors. The reduced model included Palladin expression versus only the pathologic T (T1–4, ordinal), and the full model included pathologic T and pathologic N (N0-3, ordinal), or the number of lymph nodes positive by H&E (discrete). We did not include the pathologic M factor in the models as only 21 subjects had detectable distant organ metastasis (M1), this exclusion resulted in the use of 999 M0 samples for this analysis. Standard residuals of the reduced model were calculated to display the association results (Supplementary Fig. 7). All of the statistical analyses and plots were performed using R programming language.

We gained access to the TCGA controlled data via 'The database of Genotypes and Phenotypes' (dbGaP) to retrieve rs1071738 genotypes (that is, germline). Genotype calls were available for 1,015 subjects (1,011 with normal/tumour pair) from the Affymetrix Genome-Wide Human SNP Array 6.0 (SNP_A-2089440) level 2 data. However, only 460 samples remained after excluding non-Caucasians (∼20%) and samples with missing mRNA and/or miRNA expression levels (∼200 subjects). We estimated the power of this sample size to be insufficient (<30%) by using the QUANTO software package^57^ (frequency set as 40%, and standardized effect-size as 0.1, typical for SNPs based on genome-wide association studies).

### Synthesis of miRNA-Gold nanoparticles

Bare AuNPs, with an average diameter of ≈40 nm (≈7.15E+10 nanoparticles per ml) and an SPR peak at 530 nm (extinction coefficient 8.42E+09 M ^−1^ cm^−1^, MW 3.91E+08 g mol^−1^, surface area 5.03+03 nm^2^) were purchased from Cytodiagnostics. Functionalization of PEGylated gold nanoparticles was carried out using commercial hetero-functional PEG functionalized with a 30% saturated surface of α-Mercapto-ω-carboxy PEG solution (HS-C_2_H_4_-CONH-PEG-O-C_3_H_6_-COOH, MW. 3500 Da, Sigma) as described elsewhere^58^,^59^. The 30% of saturated PEG layer allows the incorporation of additional thiolated components, such as the thiolated DNA-hairpin-Quasar 705 nm and the tiolated-oligo-BHQ2 quencher. Briefly, 10 nM of the bare-gold nanoparticles were mixed with 0.006 mg ml^−1^ of PEG solution in an aqueous solution of SDS (0.028%). After this, the mixture was incubated for 16 h at room temperature. Excess PEG was removed by centrifugation (15,000 × r.p.m., 30 min, 4 °C).

The pentapeptide CREKA (Cys-Arg-Glu-Lys-Ala), with no modifications at C- and N-terminals, was coupled to the PEG-AuNPs by a carbodiimide chemistry assisted by N-hydroxisuccinimide (EDC/NHS coupling reaction) between the carboxylated PEG terminal and the primary amine groups of the peptide. CREKA is a tumour-homing pentapeptide that specifically homes to fibrin-fibronectin complexes abundantly expressed in tumour microenvironments and specifically binds to 4T1 breast cancer cells. Briefly, 10 nM of nanoparticles-PEG, 1.98 mg ml^−1^ N-hydroxysulfosuccinimide (sulfo-NHS, Sigma) and 500 μg ml^−1^ EDC (1-Ethyl-3-(3-dimethylaminopropyl)carbodiimide, Sigma) were incubated in 10 mM MES (2-(N-morpholino)ethanesulfonic acid, Sigma) at pH 6.2 and allowed to react for 30 min to activate the carboxylic groups. After this, activated nanoparticles were washed once with 10 mM MES, pH 6.2 and used immediately. CREKA peptide was added to the mixture at a final concentration of 3 μg ml^−1^ and allowed to react for 16 h at 25 °C. After this period, the nanoparticles were centrifuged at 20.000 × g for 30 min at 4 °C and washed three times with Mili-Q water. The CREKA quantification was achieved using the Pierce BCA Protein Assay kit (Thermo Scientific) according to manufactures' instructions. Briefly, 0.025 ml of each standard and unknown sample (the supernatants) was mixed with 0.2 ml of the BCA Working Reagent (50:1, BCA reagent A:BCA reagent B) to each tube. The reaction mixture was incubated at 60 °C for 30 min. After this period the tubes were cooled down to room temperature and the absorbance measured at 562 nm. The standard curve was used to determine the CREKA concentration of each unknown sample (supernatant).

After the peptide conjugation, thiolated miRNAs (miR-96, miR-182 and a scrambled miR from Dharmacon) were dissolved in 1 ml of 0.1 M DTT, extracted three times with ethyl acetate, and further purified through a desalting Illustra NAP-5 column Sephadex G-25 DNA grade (GE Healthcare) according to the manufacturer's instructions. The purified thiolated miRNAs were incubated at a concentration of 10 μM, with an RNase-free solution of the peptide-PEG-AuNPs (10 nM) containing 0.08% SDS. Subsequently, the salt concentration was increased from 0.05 to 0.3 M NaCl with brief ultrasonication following each addition to increase the coverage of oligonucleotides on the nanoparticle surface. After functionalization during 16 h at 4 °C, the particles were purified by centrifugation (20,000*g*, 20 min, 4 °C), and re-suspended in diethyl pyrocarbonate (DEPC)-water. This procedure was repeated 3 times. The number of miRNA strands per nanoparticle was quantified using a Quant-iT RiboGreen RNA Assay Kit, which is one of the most sensitive detection dyes for the quantitation of RNA in solution, with linear fluorescence detection in the range of 1–200 ng of RNA. The standard curve was used to determine the miRNA concentration of each unknown sample (supernatant). A table summarizing the quantification of all these biomolecules can be found in Supplementary Fig. 12E.

### *In vitro* miR-nanoparticles delivery

4T1 cells stably expressing mCherry were seeded at a density of 1 × 10^5^ cells per well in 24-well plates and grown for 24 h before incubation of nanoparticles (5 nM). On the day of incubation, the cells were ∼50% confluent. For confocal microscopy, cells were fixed with 4% paraformaldehyde in PBS for 15 min at 37 °C and stained with DAPI to allow nuclear staining and finally mounted in ProLong Gold Antifade Reagent (Invitrogen). Images of cells were taken with a Nikon A1R Ultra-Fast Spectral Scanning Confocal Microscope.

### Development of orthotopic breast cancer mice model

Tumours in the mammary fat pad were induced in BALB/c (AnNCrl, 6 weeks, Charles River) female mice by injection of 1 × 10^6^ 4T1 cells stably expressing mCherry, suspended in 50 μl of HBBS (Lonza) solution. For determination of tumour growth, individual tumours were measured using caliper and tumour volume was calculated by: tumour volume (mm^3^)=width × (length^2^)/2. Treatments began when tumour volume reached about 100 mm^3^. All experimental protocols were approved by the MIT Animal Care and Use Committee and were in compliance with NIH guidelines for animal use.

### Nanoparticles-hydrogel scaffold synthesis and implantation

Tagged hydrogel scaffolds were developed by mixing equal parts of dendrimer amine of 12.5% solid content (Dendritech Inc.) and dextran aldehyde 5% solid content (Sigma-Aldrich) with 0.25% dextran (Sigma-Aldrich) to form 6 mm pre-cured disks. For doped scaffolds, miRNA-nanoparticles (10 nM) and cisplatin (30 μM, Sigma-Aldrich) were added to the dendrimer solution before hydrogel formation. All solutions were filtered through a 0.22 μm filter before hydrogel formation for *in vivo* implantation. Pre-cured disks of scaffold with nanoparticles were formed and implanted subcutaneously on top of the fat mammary tumour in BALB/c mice.

### Primary tumour growth and metastasis monitoring

Non-invasive longitudinal monitoring of tumour progression was followed by scanning mice with the IVIS Spectrum-bioluminescent and fluorescent imaging system (Xenogen XPM-2 Corporation) from mice bearing mammary tumours from 4T1 cells (*n*=5 animals per treated group). Fifteen minutes before imaging, mice were intraperitoneally injected with 150 μl of D-luciferin (30 mg ml^−1^, Perkin Elmer) in DPBS (Lonza). Whole-animal imaging was performed at the indicated time points. Assessment of *in vivo* toxicity via mouse body weight evaluation was performed on all the animal groups during 27 days after tumour induction and 20 days after hydrogel implantation. Micro-CT images of the lungs were performed in an eXplore CT120-whole mouse MicroCT (GE Healthcare) at days 13, 20 and 27 after tumour induction. Histological sections of the tumours (*n*=5) were stained with haematoxylin and eosin, and for IHC analysis the tumours (*n*=5) were stained with anti-Ki67 antibody (Abcam ab15580, dilution 1:200), anti-Vinculin (Sigma cat#v4139, dilution 1:50) or anti-Palladin (Proteintech, cat#10853-1-AP, dilution 1:50) primary antibodies.

### Statistics

We performed all statistical analyses with Student's *t*-test unless noted otherwise. Results are represented as mean±s.e.m. unless noted otherwise. No animal or sample was excluded from the analysis. The *P* values are *P<0.05, **P<0.01 and ***P<0.005.

### Data availability

The open-access TCGA data that support the findings of this study are available from https://gdc-portal.nci.nih.gov/, and the controlled-access via the Database of Genotypes and Phenotypes (dbGaP) available from http://www.ncbi.nlm.nih.gov/sites/entrez?db=gap, study access phs000178.v9.p8. The authors declare that all other data supporting the findings of this study are available within the article and its supplementary files or from the authors on a reasonable request.

## Additional information

**How to cite this article:** Gilam, A. *et al*. Local microRNA delivery targets Palladin and prevents metastatic breast cancer. *Nat. Commun.* 7:12868 doi: 10.1038/ncomms12868 (2016).

## Supplementary Material

Supplementary Information Supplementary Figures 1-16, Supplementary Tables 1-3, Supplementary Note 1 and Supplementary References.

Supplementary Dataset 1 List of SNPs located in miRNA target sites on 3'UTR of ‘breast cancer genes'

## Figures and Tables

**Figure 1:**
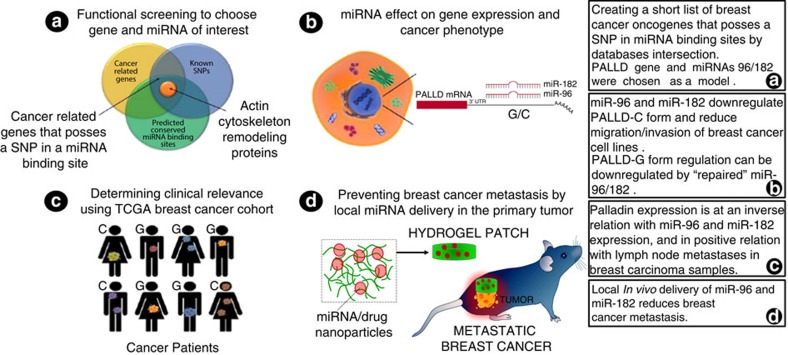
Schematic diagram of a stepwise omic-data integration which informs the *in vivo* therapeutic approach. Local miRNA delivery to the primary tumour is used to prevent breast cancer metastasis. To examine the effect of genomic alterations on breast cancer, we performed a stepwise omic-data integration to generate a narrow list of potentially functional variants for cancer metastasis, in which we intersect two datasets: TargetScan and dbSNP. Using this approach, we identified 20 SNPs, which are located at miRNA-binding sites in regulatory 3′-UTR regions of genes that are related to breast cancer (in particular those involved in cytoskeleton organization) (**a**). Our *in vitro* studies reveal miR-96 and miR-182 as key regulators of breast cancer cell motility via regulation of Palladin, which encodes a cytoskeletal protein that is required for organizing the actin cytoskeleton (**b**). Clinical data analysis supports clinical relevance in a human breast cancer cohort (**c**). Our data corroborate the role of miRNAs in metastasis regulation, and demonstrate that *in vivo* local delivery of miR-96 and miR-182 to the primary tumour can serve as a potential treatment to prevent breast cancer metastasis (**d**).
