# Supplementary material for: Local microRNA delivery targets Palladin and prevents metastatic breast cancer
Source: Nat Commun. 2016 Sep 19;7:12868. doi: 10.1038/ncomms12868 (PMC5031803; doi:10.1038/ncomms12868)
Supplement: Supplementary Information — Supplementary Figures 1-16, Supplementary Tables 1-3, Supplementary Note 1 and Supplementary References. [file ncomms12868-s1.pdf]

Supplementary Information

SUPPLEMENTARY FIGURES:

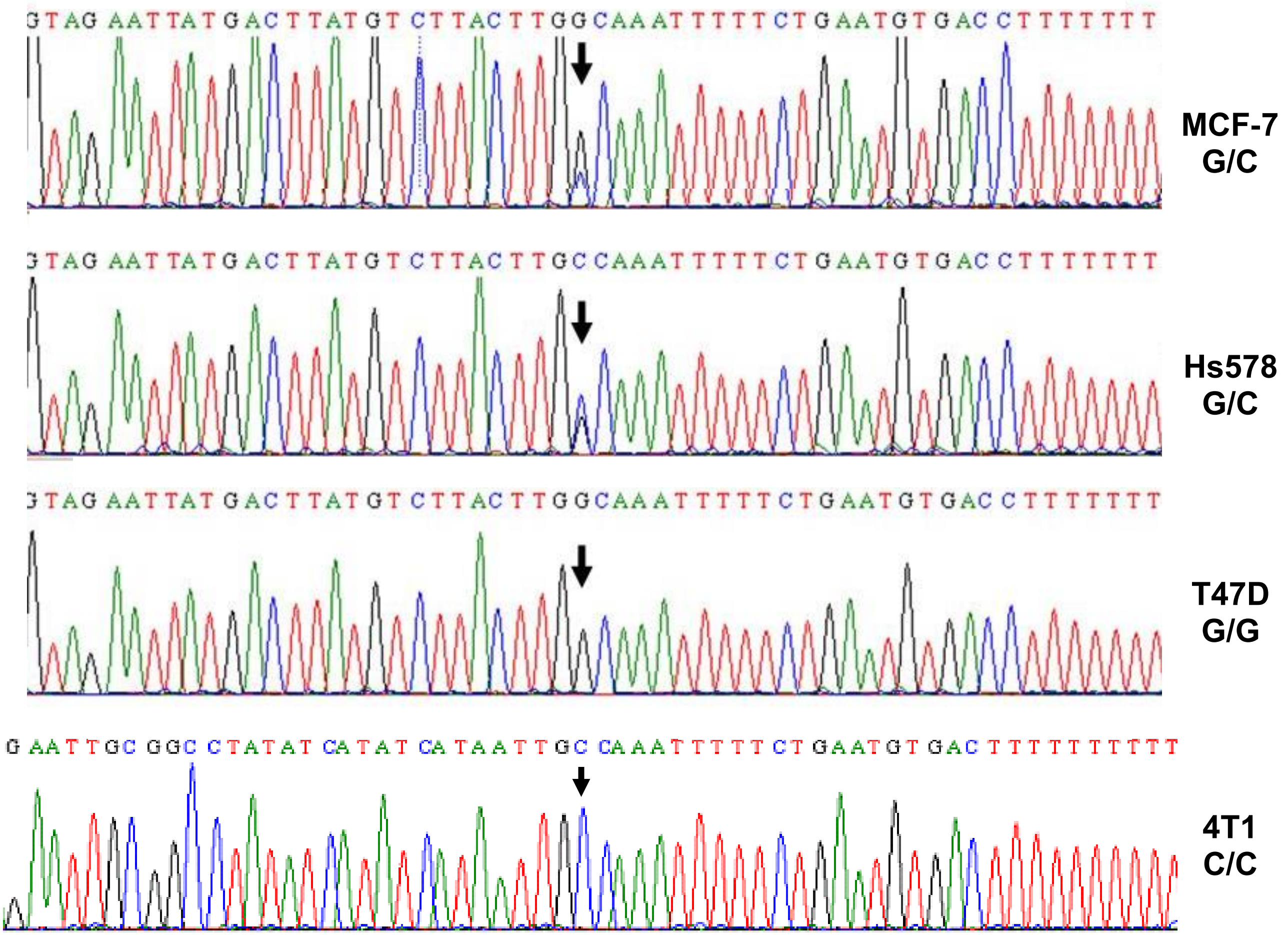

Supplementary Figure 1. rs1071738 SNP genotype of breast cancer cell lines. Sanger sequencing of regions flanking the rs1071738 SNP position on the *PALLD* 3'UTR illustrating that the human breast cancer cell lines MCF-7 and Hs578, are heterozygous for the SNP, possessing one G allele and one C allele, whereas T47D human breast cancer cells are homozygous for the G allele. The 4T1 murine breast cancer cell line was found to be homozygous for the C allele.

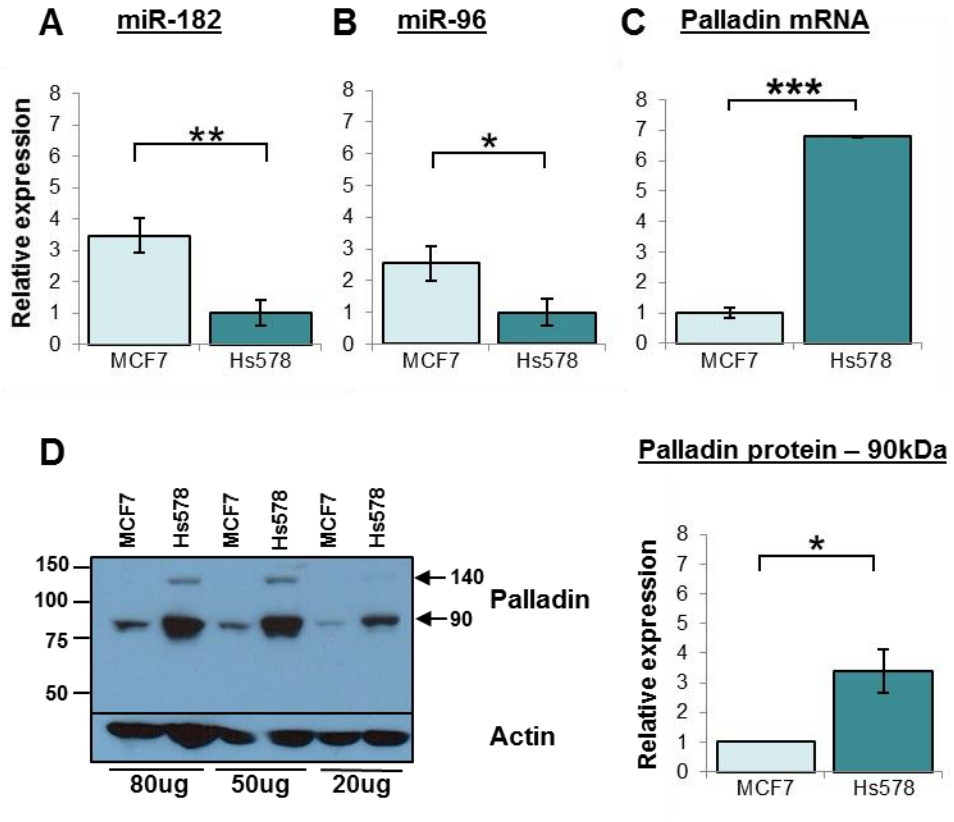

Supplementary Figure 2. miR-182, miR-96 and Palladin expression levels in MCF-7 and Hs578 breast cancer cell lines. RNA was extracted from MCF-7 and Hs578 cell lines, and the expression levels of hsa-miR-182 (A), hsa-miR-96 (B) and Palladin mRNA (C) were assayed by qRT-PCR. miRNAs levels were normalized to U6 and mRNA expression levels were normalized to GAPDH levels. (D) Palladin expression (isoform 3, 140kDa and isoform 4, 90kDa – see arrows) in MCF-7 and Hs578 cells. Expression levels were determined using Western blot analysis and were normalized to Actin levels. Palladin Isoform 3 was undetected in MCF-7 cells. Palladin isoform 1 was undetected in both cell lines. The relative expression levels of Palladin isoform 4 (90kDa) are presented on the right. Values are presented as mean ± SEM (n=3, Student's t-test, $*p<0.05$, $**p<0.01$, $***p<0.005$).

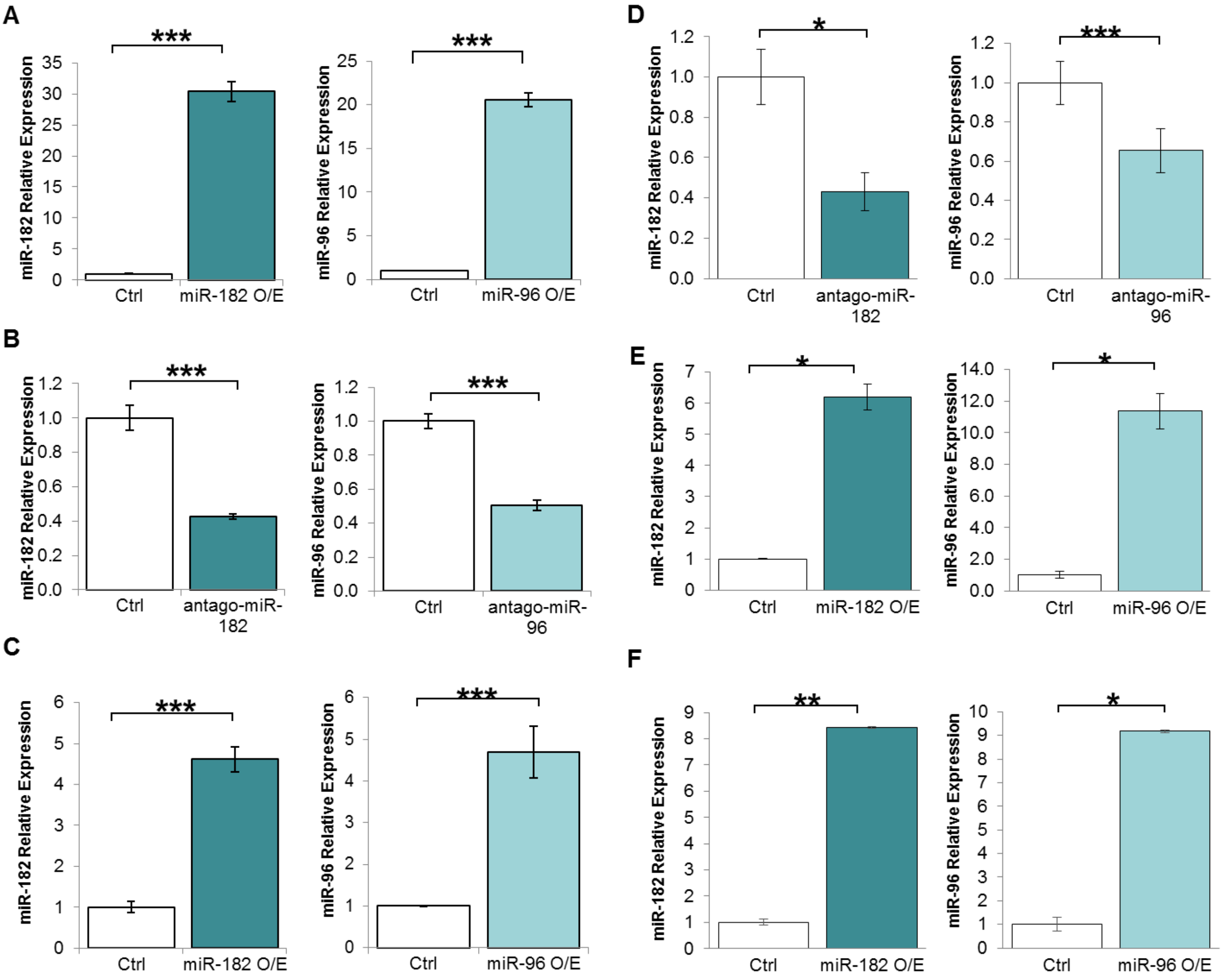

Supplementary Figure 3. Changes in miRNA levels following overexpression or downregulation. (A) RNA was extracted from Hs578 cells 24 hours following overexpression of hsa-miR-182, hsa-miR-96, or pcDNA3 control plasmid (Ctrl). (B) RNA was extracted from MCF-7 cells 24 hours following transfection with antago-hsa-miR-182, antago-hsa-miR-96, or scrambled control (Ctrl). (C) RNA was extracted from 4T1 cells 2-3 weeks following infection of mmu-miR-182, mmu-miR-96, or scrambled plasmid (Ctrl). (D) RNA was extracted from 4T1 cells 48 hours following transfection with antago-mmu-miR-182, antago-mmu-miR-96, or scrambled control (Ctrl). (E) RNA was extracted from 4T1 Palladin KD cells (stably expressing Palladin shRNA), 48 hours following overexpression of mmu-miR-182, mmu-miR-96, or scrambled plasmid (Ctrl). (F) RNA was extracted from 4T1 control cells (infected with scrambled control shRNA) 48 hours following overexpression of mmu-miR-182, mmu-miR-96, or scrambled plasmid (Ctrl). miRNA levels were assayed by qRT-PCR, and were normalized to U6 levels. Values are presented as mean ± SEM (n=3, Student's t-test, *p<0.05, **p<0.01, ***p<0.005).

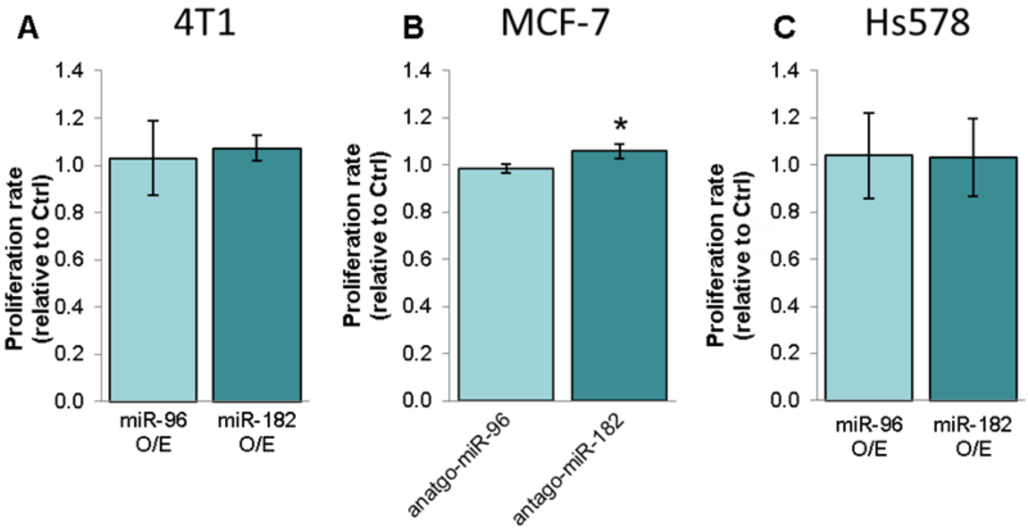

Supplementary Figure 4. Proliferation rate is not affected by miR-96/182 levels. (A and B) Proliferation rate was determined 24-48 hours following transfection with the indicated miRNAs or antago-miRs, using FITC BrdU Flow Kit (BD Biosciences). Anti FITC-BrdU and DAPI were used in order to determine the proportion of cells in each cell cycle phase. The S/G1 ratios in treated cells (4T1 cells in A and MCF-7 cells in B) are presented (n=3). (C) Proliferation rate was determined 48 hours following transfection of Hs578 cells with the indicated miRNAs using ViaLight Plus Cell proliferation and cytotoxicity assay (Lonza) (n=4). Values are presented as mean ± SEM (Paired student t-test, *$p<0.05$).

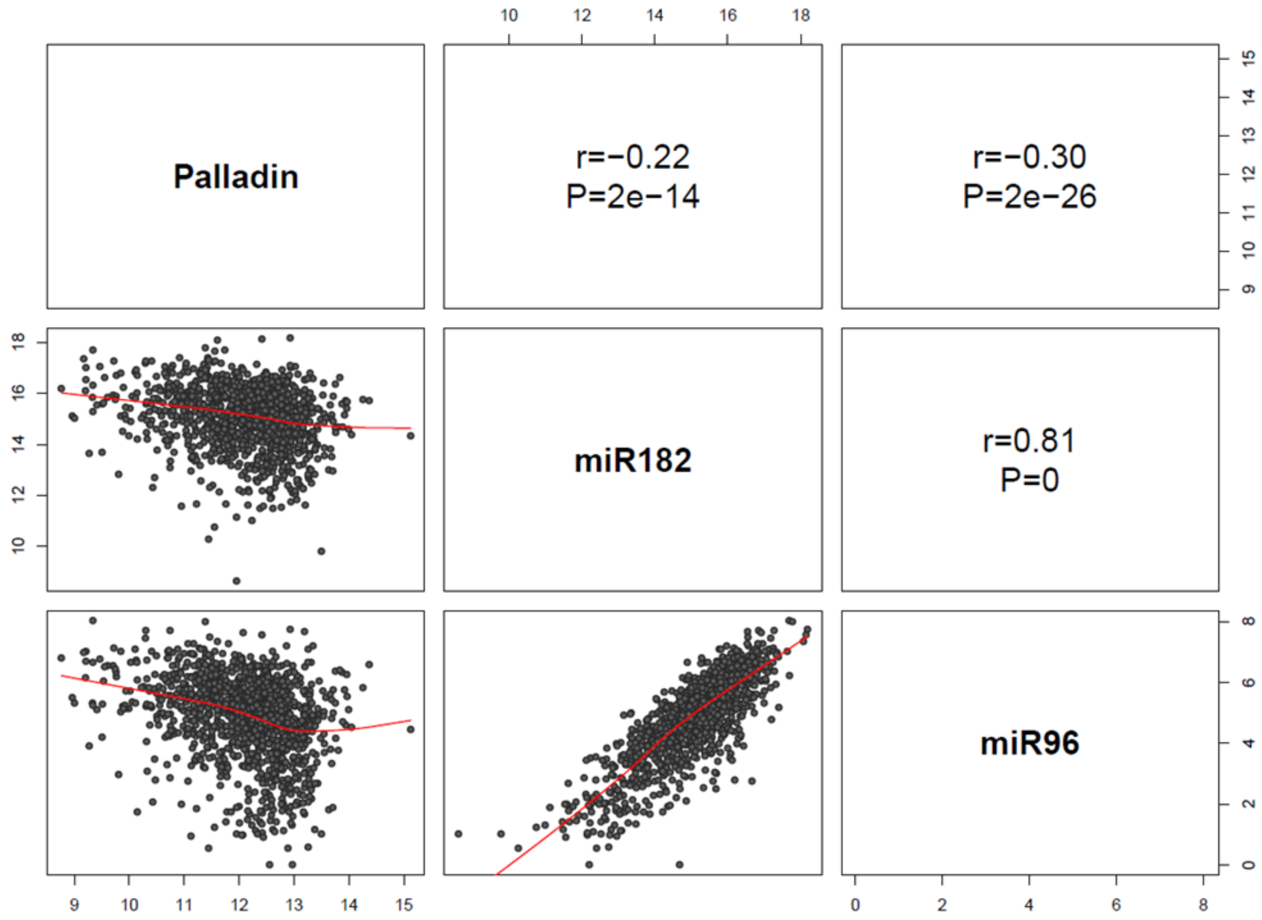

Supplementary Figure 5. Palladin and miR-96/82 are inversely correlated in human breast cancer samples. Pairwise scatterplots of Palladin and miR-96 and miR-182 normalized expression in 1,176 breast invasive carcinomas study samples from The Cancer Genome Atlas (TCGA) (lower diagonal). Correlation between transcription normalized counts was measured using the Pearson's method. The correlation coefficients and P-value (P) are shown in the upper diagonal. The expression of miR-96 and miR-182 was highly positively correlated, as expected for cluster miRNAs.

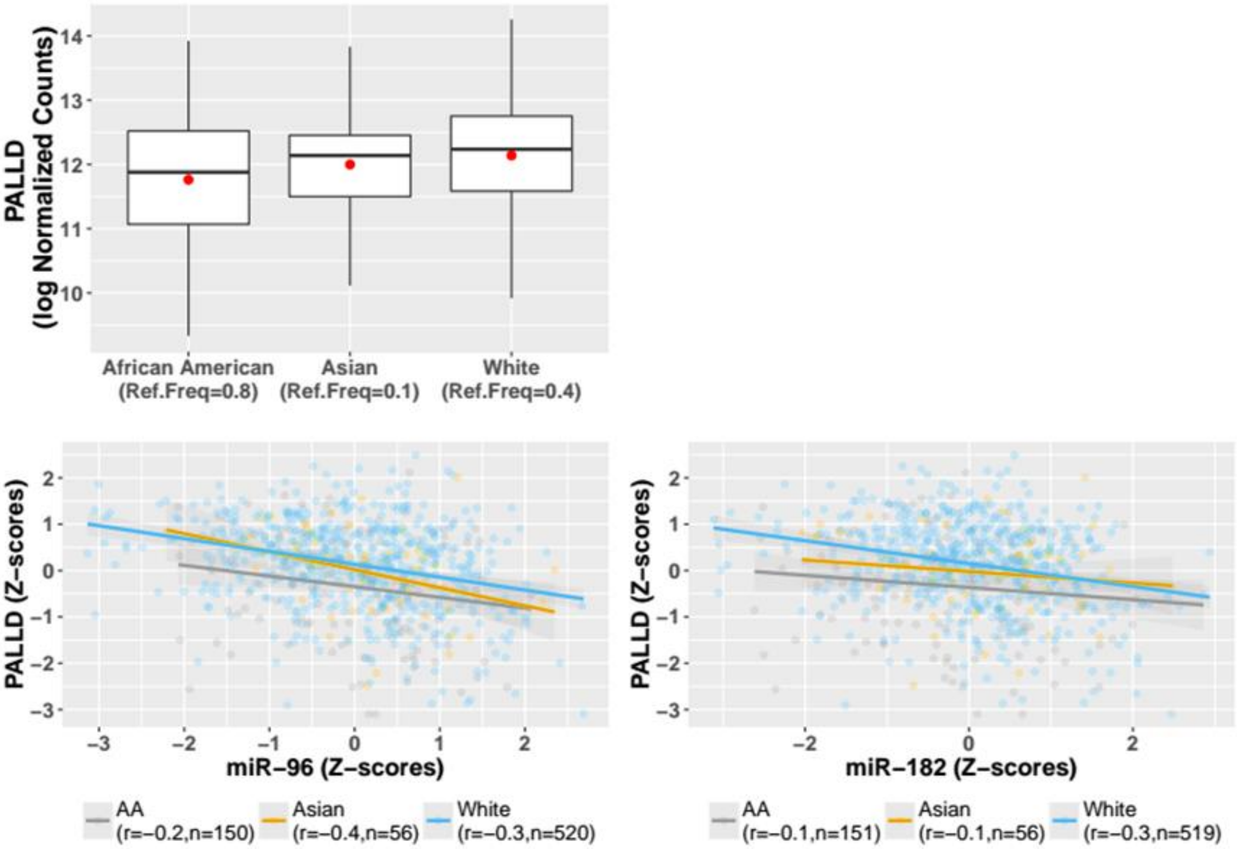

Supplementary Figure 6. Palladin expression and correlation with miR-96/82 in human breast cancer by sample ancestry. Boxplots of Palladin normalized expression levels (y-axis) by sample ancestry (x-axis) are displayed in the top panel. Ancestry-specific frequency of the reference C allele is listed in brackets. As displayed, Palladin mean expression levels were significantly lower in African-American versus White decent ($P = 3.4x10^{-6}$), in line with the C allele expression lowering effect. Scatterplots of Palladin and miR-96 (bottom left) and miR-182 (bottom right) normalized expression in TCGA breast invasive carcinomas by sample ancestry. Correlation between transcript normalized counts was measured using the Pearson's method. The correlation coefficients and sample size (n) are shown in brackets below.

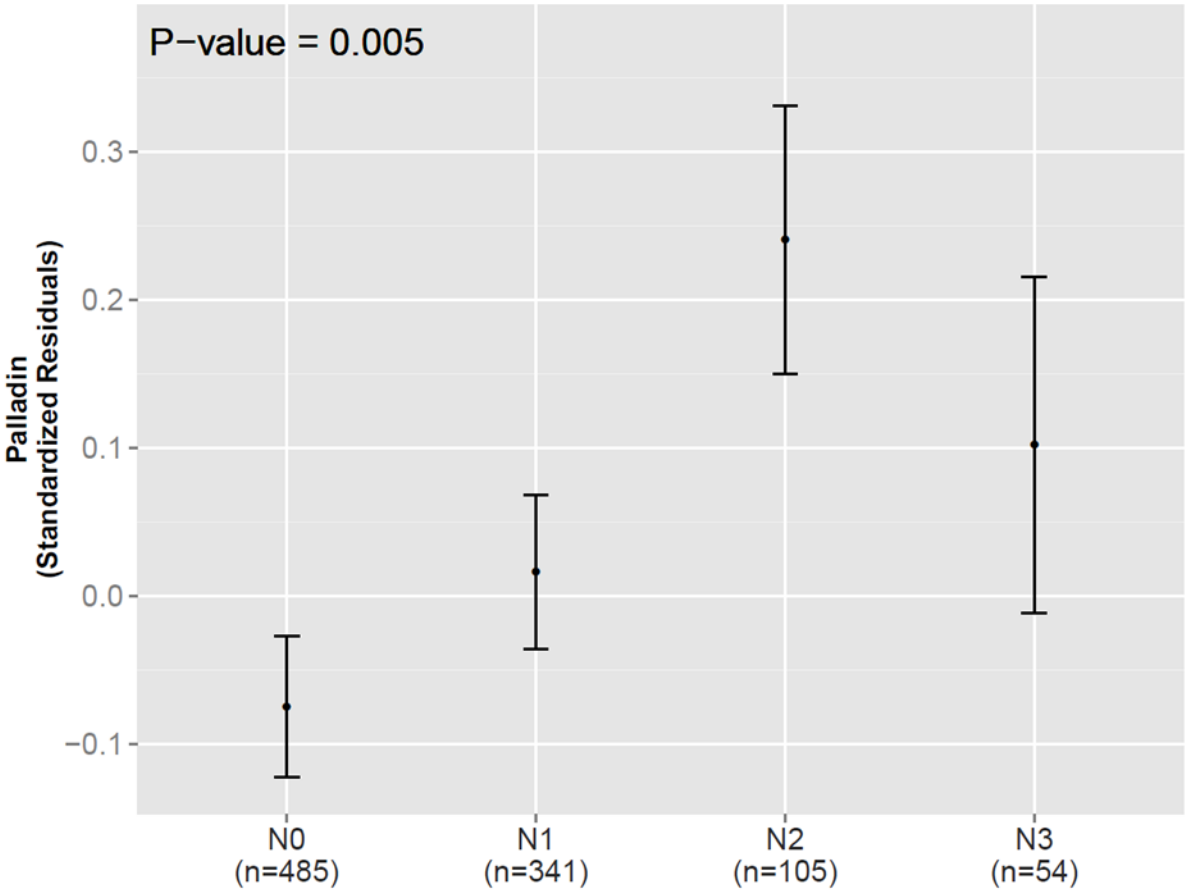

Supplementary Figure 7. Positive relationship between Palladin expression and lymph node metastatic stage in TCGA Breast invasive carcinoma (BRCA) cohort. The circles represent the estimated marginal means of the standardized Palladin residual values in the corresponding pathologic N stage group. Error bars represent the standard errors. The p value (see Table S3) was obtained by ANOVA analysis for the standardized Palladin residuals corrected for tumor size (pathologic T).

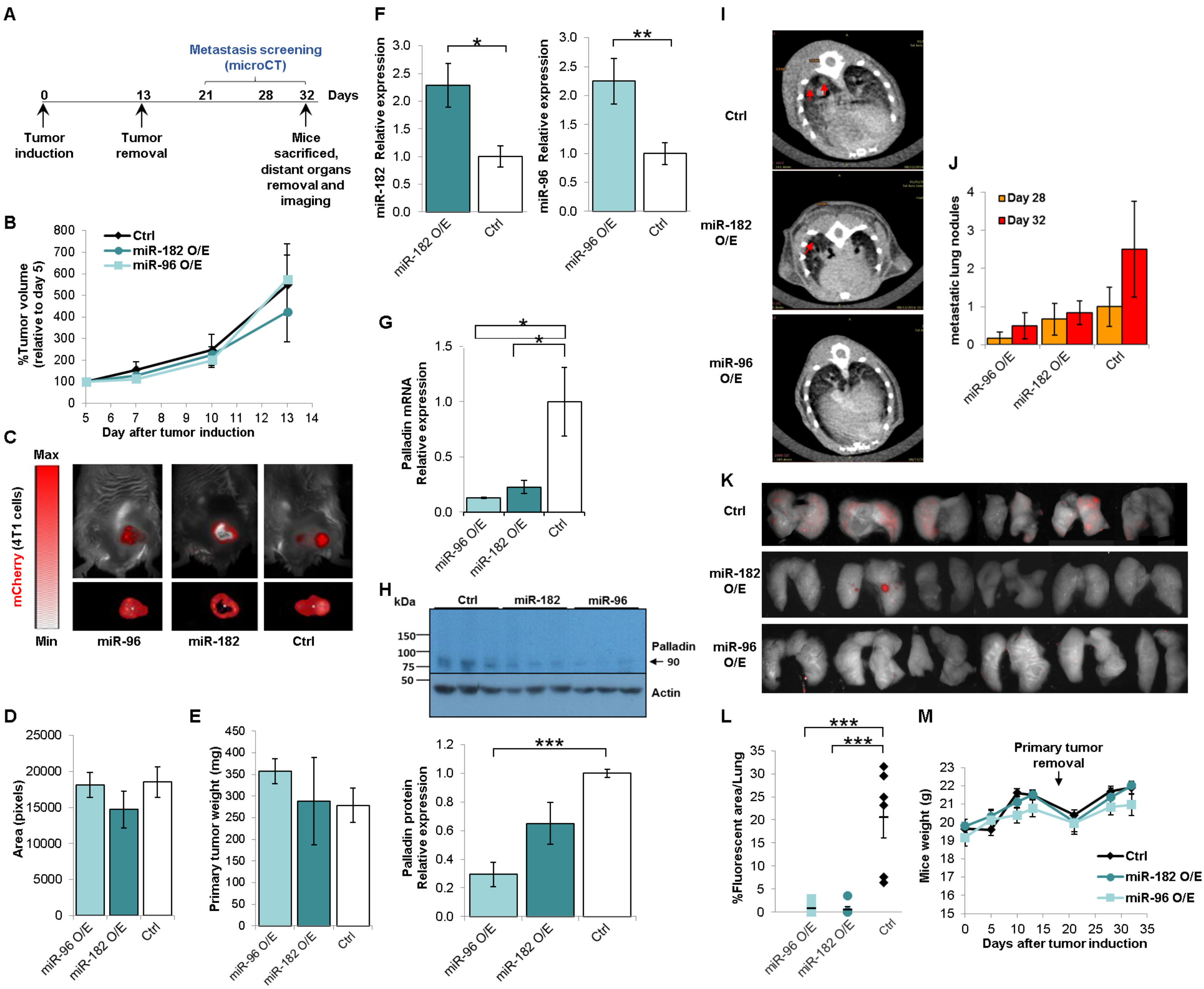

Supplementary Figure 8. miR-96 and miR-182 diminish metastatic potential *in vivo*. (A) Experiment flow: On day 0, 4T1-mCherry cells, stably expressing mmu-miR-96, mmu-miR-182 or scrambled sequence as control (Ctrl), were injected into the mammary fat pad of female mice (n=6 per group). Tumor volumes were monitored until removal on day 13. Presence of macro-metastases was assessed on days 21, 28 and 32 using the CT imaging system. Mice were sacrificed at day 32 and lungs were removed and tested for metastases presence. (B) The change in tumor volume over time (relative to day 5, when the tumors were palpable). (C) Live imaging of primary tumors. Mice were photographed by the CRI-MAESTRO imaging system on day 13, before tumor removal. The intensity of the signal is indicated by the scale. Representative photos for measurements of total fluorescent signal (upper panel) and fluorescent signal area (lower panel) are shown. (D) Quantification of fluorescent signal averaged area of primary tumors. (E) Average weight of primary tumors following removal at day 13. (F) miRNA levels in primary tumors following removal, normalized to U6 levels (n=6). Palladin mRNA (G) and Protein (H) levels in primary tumors following removal. Mouse Palladin mRNA levels were assayed by qRT-PCR and were normalized to mouse β-Actin levels (n=6). Palladin protein levels were determined by Western blot analysis and were normalized to Actin levels (n=3). (I) Representative micro-CT images of mouse lungs taken on Day 32. Arrows show the presence of macro-metastasis. (J) Quantification of metastatic lung nodules in mice for days 28 and 32. (K) *Ex vivo* fluorescent images of lungs depicting mCherry emission. (L) Quantification of lung area covered by fluorescent signal. (M) Mouse weight from inoculation day (day 0) to euthanasia (day 32). Values are presented as mean ± SEM (Student's t-test,*$p<0.05$, **$p<0.01$, ***$p<0.005$).

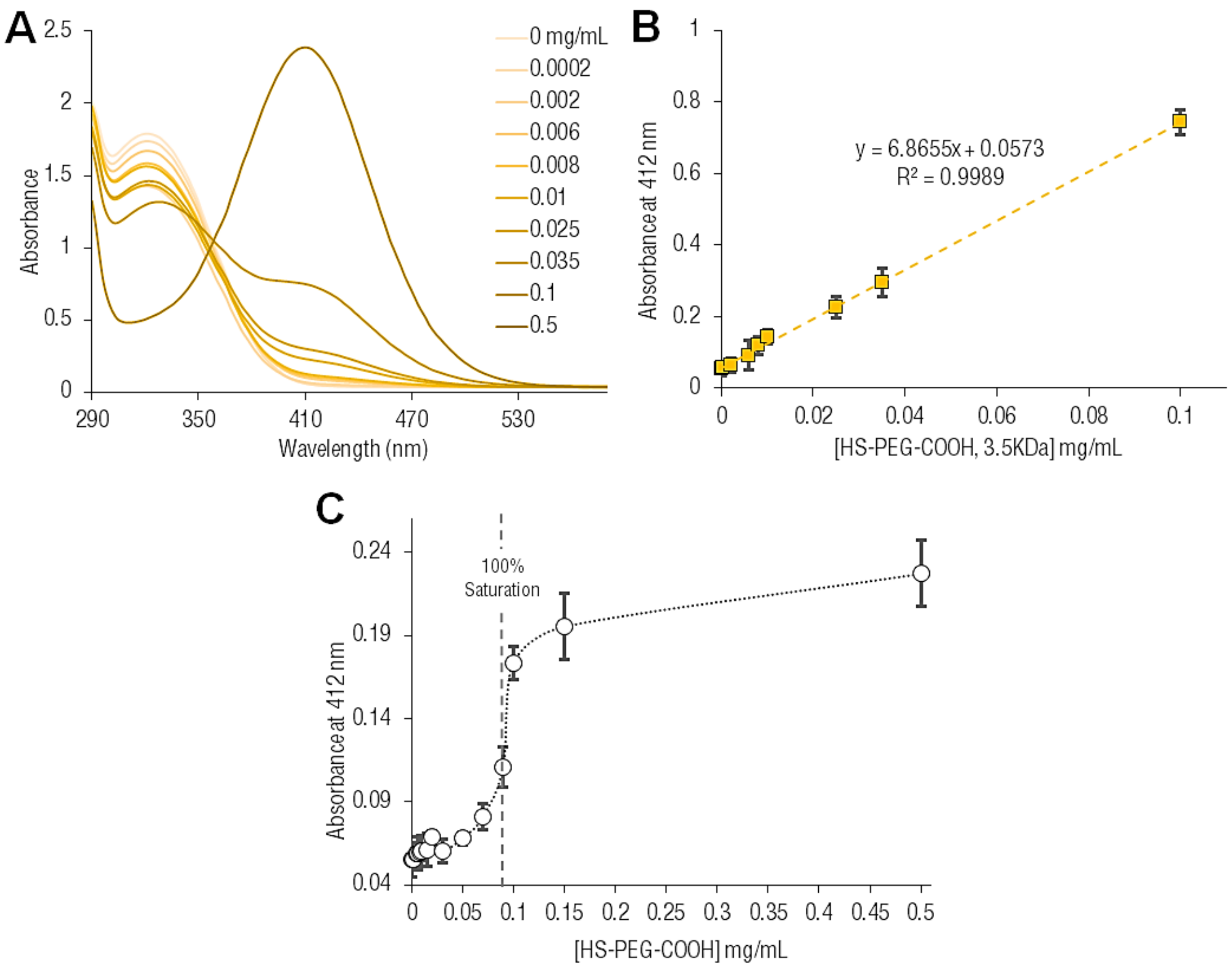

Supplementary Figure 9. PEG functionalization and quantification on gold nanoparticles. (A) Absorbance spectra of DTNB after reaction with increasing amounts (0-0.5 mg/mL) of thiolated PEG (HS-PEG-COOH, 3.5 KDa). (B) Standard calibration curve for PEG chains, whose concentration can be calculated via the following equation: Abs at 412nm = 6.8655×[PEG, mg/mL] + 0.0573, R^2=0.9989. (C) Variation of the excess of PEG thiolated chains as a function of the initial concentration in the incubation with 10 nM of AuNPs. The dashed vertical line indicates the 100% saturation, i.e. the PEG concentration above which no more PEG can be bound to the nanoparticle's surface. Values are presented as mean ± SEM.

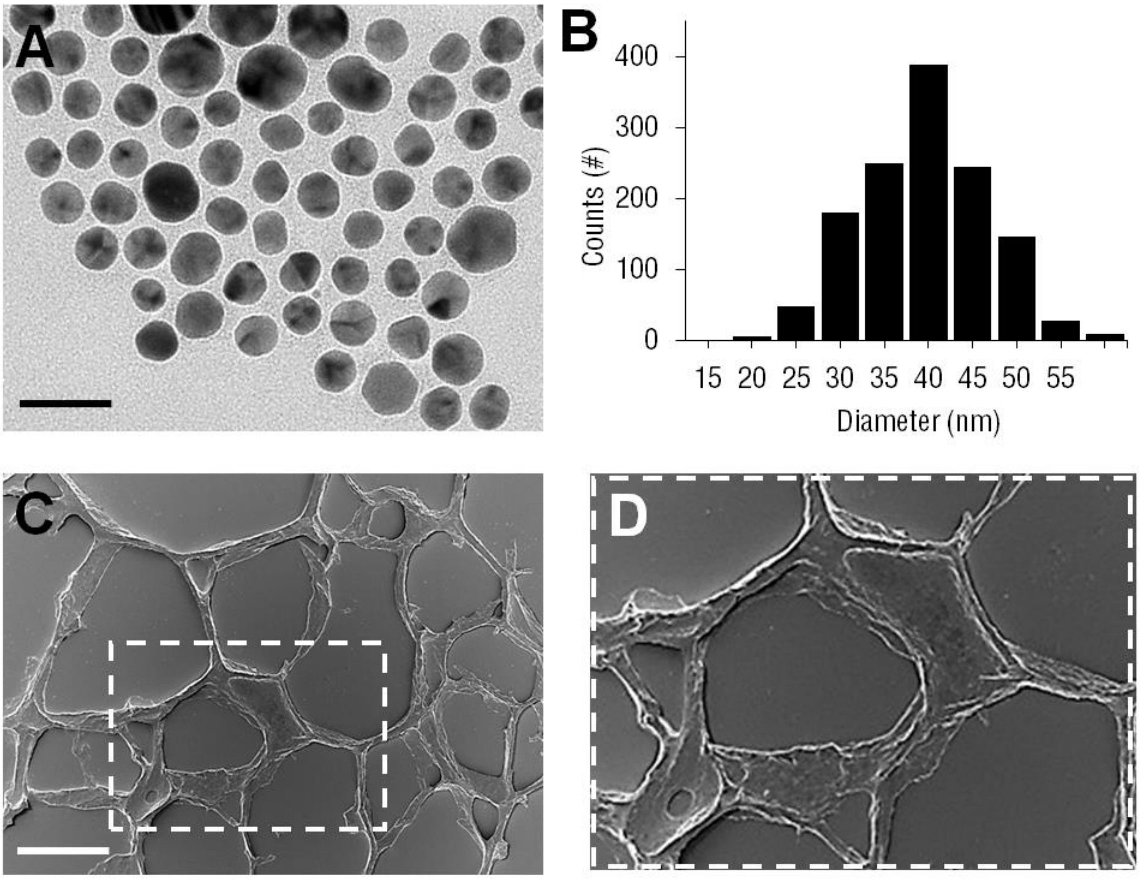

Supplementary Figure 10. Gold nanoparticles and hydrogel scaffold caracterization. (A) TEM images (scale bar = 50 nm) and (B) sizing histogram (average diameter ≈ 40 nm) of bare gold nanoparticles. (C-D) High-resolution SEM images of the 3D structure of the dextran-dendrimer hydrogel scaffolds (scale bar in C = 100 μm). The samples were coated with gold/palladium (10-15 nm thickness).

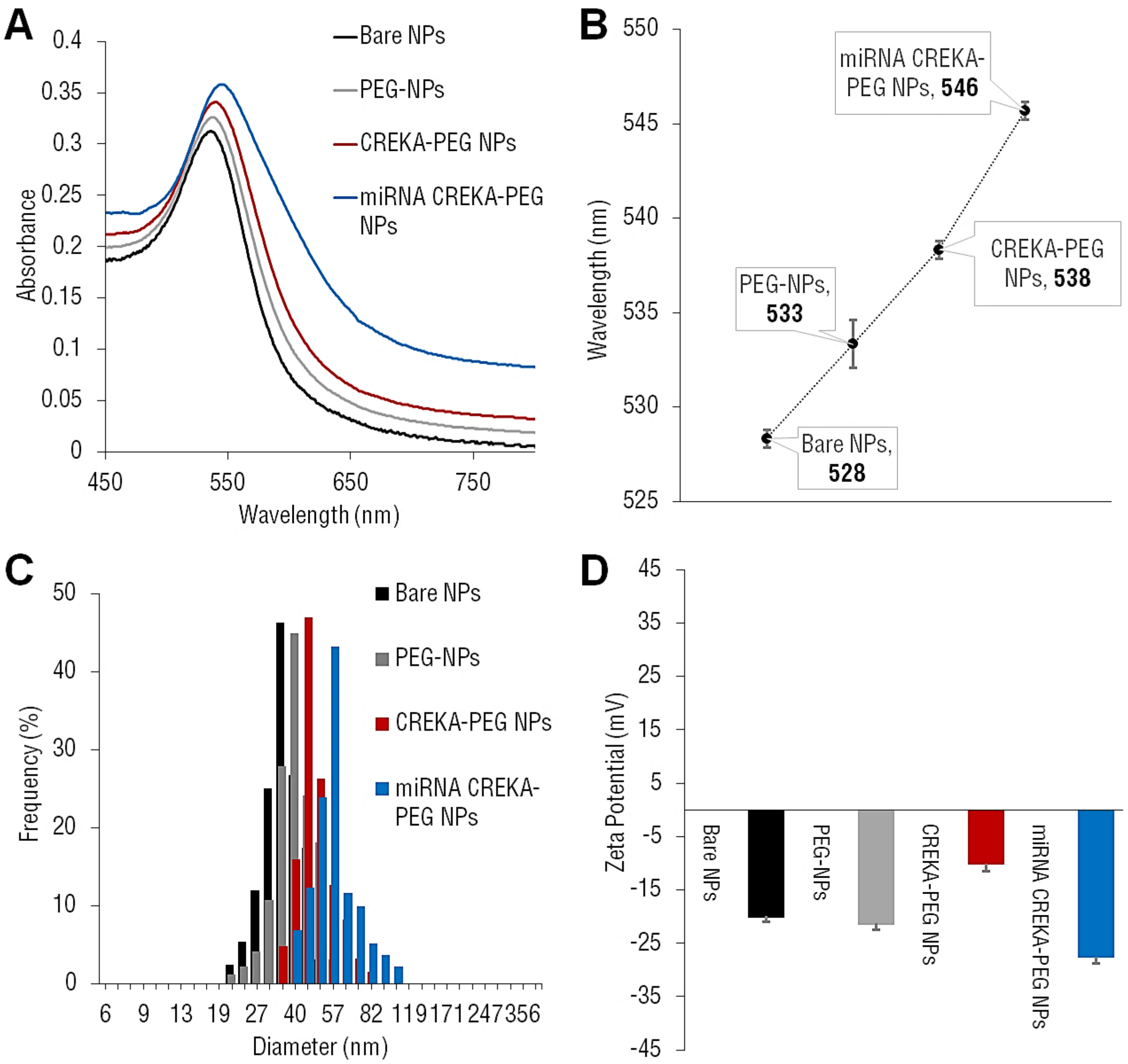

Supplementary Figure 11. Gold nanoparticles characterization. (A) Absorption band of the resulting AuNPs appeared between ~530 to ~550 nm due to the surface plasmon resonance (SPR) of the AuNPs. (B) The spatial arrangement of AuNP can be inferred by measuring the shift of a characteristic SPR band depending on size, shape, aggregation state, and medium polarity. The SPR of the AuNPs shown exhibited a red shift from 528 to 546 nm: a 5 nm shift for PEG binding, a 5 nm shift for CREKA peptide binding and an 8 nm shift for miRNA binding indicating successful binding of the several groups. (C) Dynamic light scattering (DLS) measurements with diameter distribution histograms and (D) Zeta Potential of the resulting AuNPs. All experiments were done in triplicate and values are presented as mean ± SEM.

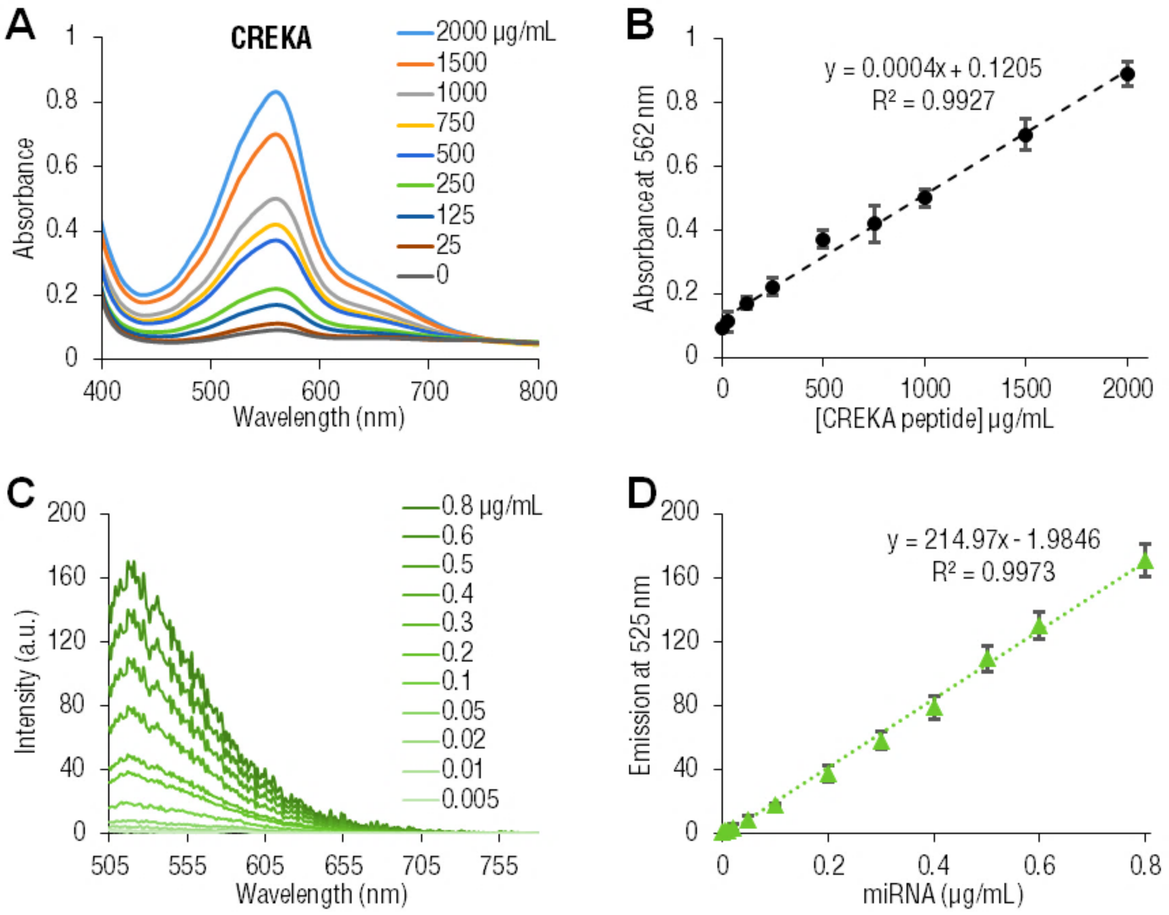

E

Nanoformulation	Size (nm)	Zeta-Potential (mV)	PEG mol per particle	CREKA peptide mol per particle	miR mol per particle
**NPs-PEG**	43.7±2.5	- 32.8±2.4	804.6±10.6	NA	NA
**NPs-PEG-Peptide**	45.8±1.8	- 25.4±4.5	804.6±10.6	146.2±3.8	NA
**NPs-PEG-Peptide miR-182**	51.0±1.6	- 30.4±2.1	804.6±10.6	146.2±3.8	248.3±5.6
**NPs-PEG-Peptide miR-96**	50.7±2.1	- 30.2±3.4	804.6±10.6	146.2±3.8	250.3±4.4
**NPs-PEG-Peptide Ctrl miR**	50.9±2.7	- 31.3±2.9	804.6±10.6	146.2±3.8	251.8±2.6

Supplementary Figure 12. Quantification of the functionalized peptide and miRNAs on the surface of the PEG-gold nanoparticles. (A) BCA assay for CREKA peptide quantification. BCA exhibits a strong absorbance at 562 nm in a near linear relationship with increasing CREKA peptide concentrations from 25 to 2000 µg/mL. (B) Standard calibration curve (Abs562nm = 0.0004×[CREKA peptide, µg/mL] + 0.1205, R^2=0.9927) for the CREKA peptide in the conditions of the EDC/NHS coupling reaction. (C-D) Linear quantitation of miRNAs (from 0.005 to 0.8 µg/mL) using the RiboGreen® RNA quantitation reagent. Samples were excited at 485 nm, and the fluorescence emission intensity was measured at 525 nm. Background fluorescence was not subtracted. (E) Table summarizing the size and charge of all nanoparticles used in this study as well as the quantification of PEG, CREKA peptide and miRNAs. All experiments were done in triplicate and values are presented as mean ± SEM.

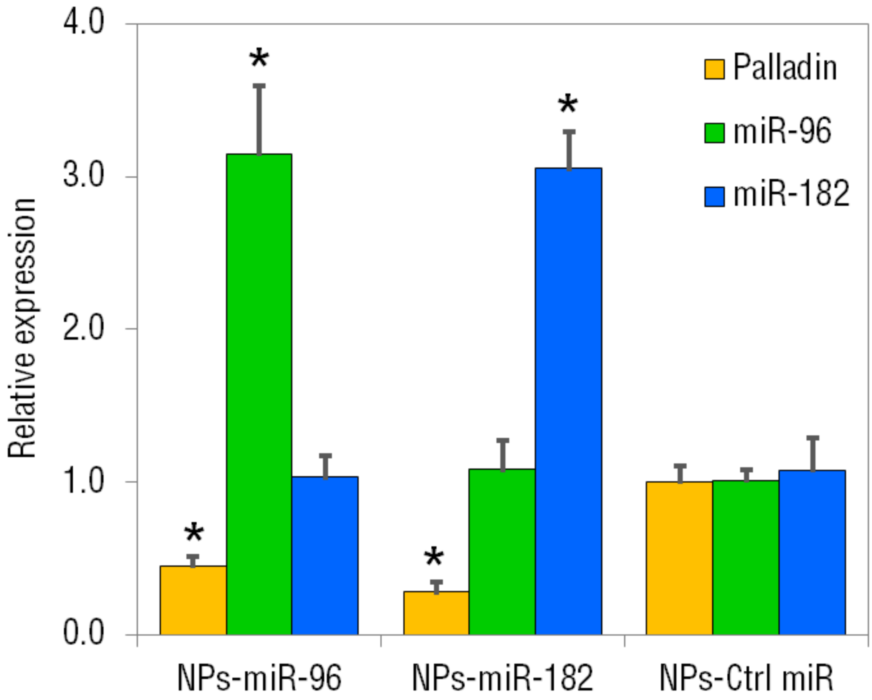

Supplementary Figure 13. Quantitative PCR (qPCR) determination of miR-96, miR-182 and Palladin mRNA expression levels in 4T1 cells treated with targeted gold NPs carrying miR-96, miR-182 or scrambled (Ctrl) miRNAs. Values are presented as mean ± SEM (n=5, Student's t-test, *p<0.05).

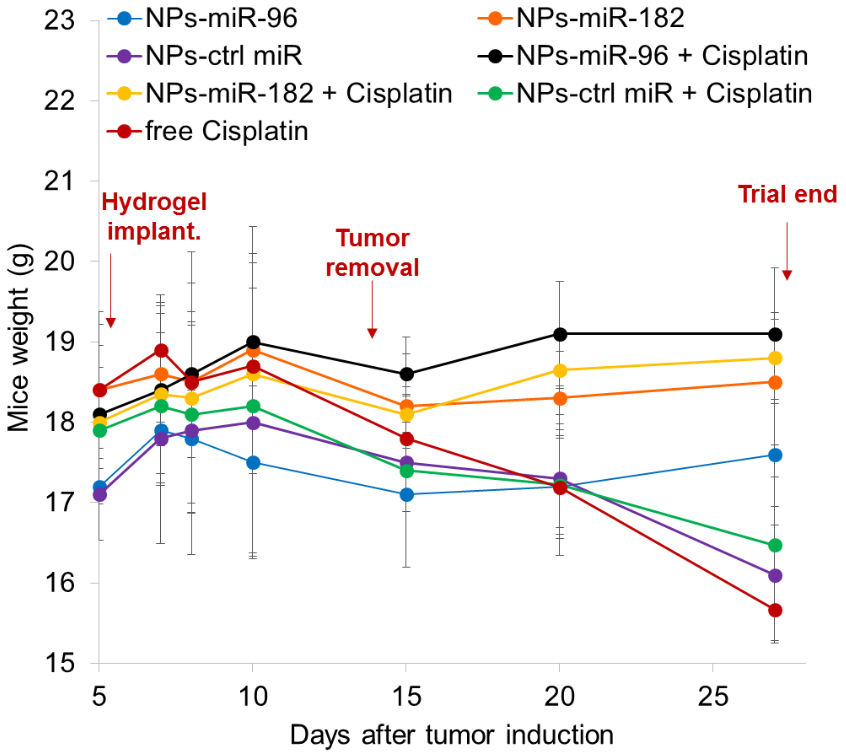

Supplementary Figure 14. The safety of hydrogel doped with targeted miR-nanoparticles was confirmed by monitoring body weight as a proxy for tolerability. Body weight assessment was performed on all the animal groups during the 27 days after breast tumor induction and 20 days after hydrogel implantation. Body weight depicted as the mean of each treatment group ± SEM (n= 5).

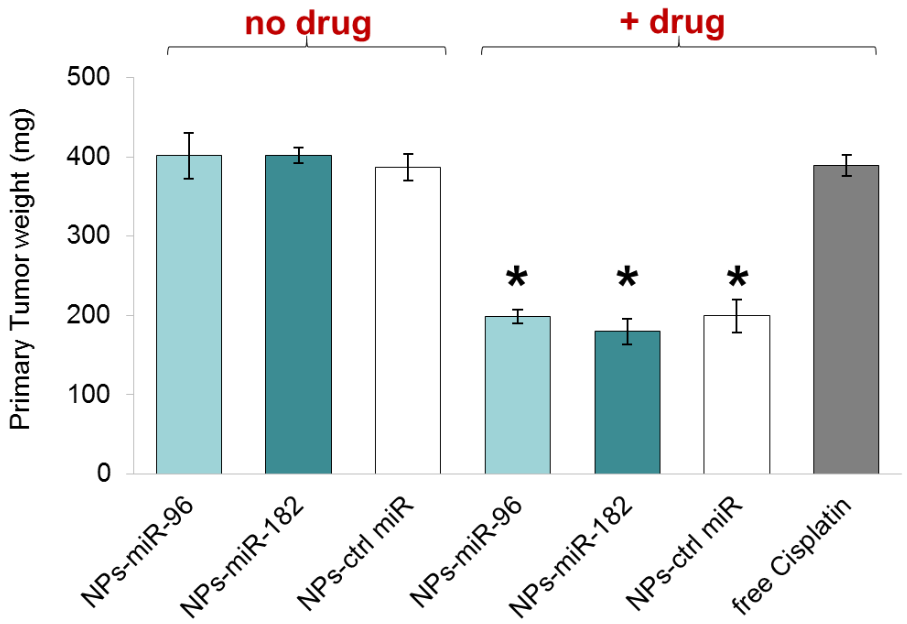

Supplementary Figure 15. Primary tumor weight following treatment with targeted miR-nanoparticles with and without cisplatin. Values are presented as mean ± SEM (n=5 per group, Student's t-test, *$p < 0.05$).

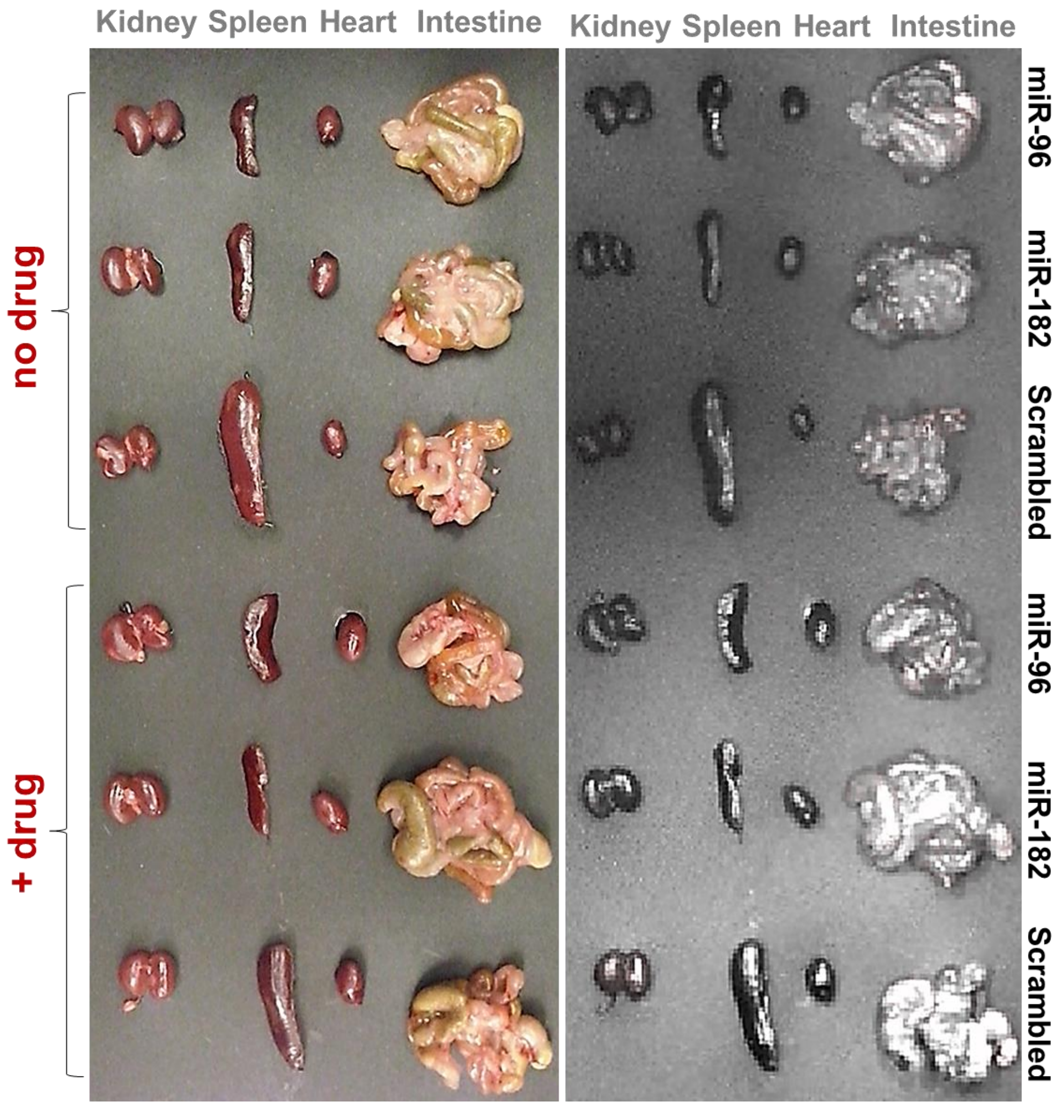

Supplementary Figure 16. *Ex vivo* fluorescent images of kidney, spleen, heart and intestine depicting mCherry emission (right panel) in treated mice.

SUPPLEMENTARY TABLES:

Supplementary Table 1. 'Actin cytoskeleton' genes - Minor allele frequencies and publication on involvement in metastasis

Gene. Symbol	Chr	SNP.rsID	SNP.Position[1]	Allele	MAF[2]	Supplementary reference #[3]	Cancer Type
PALLD	**chr4**	**rs1071738**	**169849389**	**C,G,**	**0.43**		
ROCK2	chr2	rs978906	11323276	A,G,	0.39	1-3	osteosarcoma, hepatocellular carcinoma, cholangiocarcinoma
KRT20	chr17	rs3169914	39032395	A,G,	0.37		
FGF7	chr15	rs1057636	49776957	A,C,	0.37		
FGF7	chr15	rs79465035	49777671	-,A,	0.23		
ABR	chr17	rs11247571	908502	C,T,	0.32		
MYLK	chr3	rs34709307	123331776	-,A,	0.27		
BCR	chr22	rs3876062	23658769	A,G,	0.24		
S100A8	chr1	rs3006488	153362507	A,G,	0.17	4-6	breast cancer, gastric adenocarcinoma, cervical cancer
CSF1R	chr5	rs3828609	149432863	C,T,	0.16	7	breast cancer
EPHA3	chr3	rs73139148	89530956	A,G,	0.13	8	hepatocellular carcinoma
PRKAR1A	chr17	rs8905	66527802	G,T,	0.12		
PARVA	chr11	rs11022392	12551397	A,G,	0.11	9	lung adenocarcinoma
RHOG	chr11	rs1049388	3848373	C,G,	0.08		
CCDC88A	chr2	rs17046829	55517219	C,T,	0.05		
PDGFRB	chr5	rs6674	149493535	A,G,	0.03	10	mesenchymal-like colorectal
TACC1	chr8	rs57661490	38707214	C,T,	0.01		
ACTG1	chr17	rs1140892	79477356	A,G,	0.01		
ADRA2A	chr10	rs13306145	112839999	A,G,	0.01		
BCL2	chr18	rs113207678	60794276	A,T,	0		

[1] SNP position based on the Feb. 2009 assembly of the human genome (hg19)

[2] Minor Allele Frequency (MAF) in 1000 genomes (ALL), or in dbSNP when missing from 1000 genomes

[3] Reference for publication suggesting a role in metastasis for the specified gene

Supplementary Table 2. Association of Palladin and miR-96 and miR-182 expression with lymph node metastases in The Cancer Genome Atlas (TCGA) Breast invasive carcinoma cohort

Lymph node metastases	Transcript	N	Effect(SEM)[a]	P-value[b]
Pathologic N (N0-3, ordinal)	Palladin	982	0.11±0.04	**5.20E-03**
	miR-182	957	0.06±0.04	1.22E-01
	miR-96		-0.02±0.04	5.83E-01
Number of lymph nodes (discrete)	Palladin	854	0.06±0.02	**2.61E-03**
	miR-182	835	0.02±0.02	6.42E-01
	miR-96		-0.01±0.02	2.14E-01

[a] The effect size represents the proportion of 1 SD change in standardized transcript residuals after adjustment for tumor stage (Pathologic N)

[b] P-values were calculated using ANOVA

Supplementary Table 3. SYBR green RT-PCR primers for mRNA quantification

Primer name	Sequence
hPalladin-For	AACCGAGCAGGACAGAAC
hPalladin-Rev	TGGTGGCACTCCCAATAC
hGAPDH-For	AGCCACATCGCTGAGACA
hGAPDH-Rev	GCCCAATACGACCAAATCC
mPalladin-For	AGCATGCACCAGGATAATCA
mPalladin-Rev	CAGGACACAATGCCTGCTT
m β-Actin-For	ACCAGAGGCATACAGGGACA
m β-Actin-Rev	CTAAGGCCAACCGTGAAAAG

SUPPLEMENTARY NOTES

Supplementary Note 1. Further Acknowledgments

The Shomron team is funded by: Israel Cancer Research Fund (ICRF), Research Career Development Award (RCDA); Wolfson Family Charitable Fund; Claire and Amedee Maratier Institute for the Study of Blindness and Visual Disorders; I-CORE Program of the Planning and Budgeting Committee, The Israel Science Foundation (grant number 41/11); The Israeli Ministry of Defense, Office of Assistant Minister of Defense for Chemical, Biological, Radiological and Nuclear (CBRN) Defense; Saban Family Foundation, Melanoma Research Alliance; Binational Science Foundation (BSF); Israel Cancer Research Fund (ICRF) Acceleration Grant; Israel Cancer Association (ICA); donation from the Kateznik K. Association Holocaust; Margot Stoltz Foundation by the Faculty of Medicine grants of Tel Aviv University; The Varda and Boaz Dotan Research Center in Hemato-Oncology, Idea Grant; 'Lirot' Association and the Consortium for Mapping Retinal Degeneration Disorders in Israel; Interdisciplinary grant of the Israeli Ministry of Science, Technology and Space on the Science, Technology and Innovation for the Third Age; The Edmond J. Safra Center for Ethics at Tel Aviv University; Checkpoint Institute for Information Security; Joint Core Program of Research on the Molecular Basis of Human Disease, Shabbetai Donnolo Fellowships supported by the Italian Ministry of Foreign Affairs; The Edmond J. Safra Center for Bioinformatics at Tel Aviv University; Israel Science Foundation (No. 1852/16).

We thank the Swanson Biotechnology Center at the Koch Institute for Integrative Cancer Research at Massachusetts Institute of Technology (MIT) for assistance with animal experiments and facilities, especially the microscopy and histology cores. We also acknowledge that all qPCR experiments done in the KI Genomics Core/ MIT BioMicro Center are funded by the NIH and supported in part by the Koch Institute Support Grant P30-CA14051 from the National Cancer Institute and by the National Institute of Environmental Health Sciences of the NIH under award P30-ES002109.

SUPPLEMENTARY REFERENCES:

1. Huang, D. *et al.* Rock2 promotes the invasion and metastasis of hepatocellular carcinoma by modifying MMP2 ubiquitination and degradation. *Biochem. Biophys. Res. Commun.* **453,** 49–56 (2014).
2. Peng, F. *et al.* Direct targeting of SUZ12/ROCK2 by miR-200b/c inhibits cholangiocarcinoma tumourigenesis and metastasis. *Br. J. Cancer* **109,** 3092–3104 (2013).
3. Wang, W., Zhou, X. & Wei, M. MicroRNA-144 suppresses osteosarcoma growth and metastasis by targeting ROCK1 and ROCK2. *Oncotarget* **6,** 10297–10308 (2015).
4. Choi, J.-H. *et al.* Identification of S100A8 and S100A9 as negative regulators for lymph node metastasis of gastric adenocarcinoma. *Histol. Histopathol.* **27,** 1439–1448 (2012).
5. Qin, F. *et al.* S100A8/A9 induces apoptosis and inhibits metastasis of CasKi human cervical cancer cells. *Pathol. Oncol. Res. POR* **16,** 353–360 (2010).
6. Vrakas, C.-N. *et al.* The Measure of DAMPs and a role for S100A8 in recruiting suppressor cells in breast cancer lung metastasis. *Immunol. Invest.* **44,** 174–188 (2015).
7. Patsialou, A. *et al.* Autocrine CSF1R signaling mediates switching between invasion and proliferation downstream of TGFβ in claudin-low breast tumor cells. *Oncogene* **34,** 2721–2731 (2015).

8. Lu, C.-Y. *et al.* High levels of EphA3 expression are associated with high invasive capacity and poor overall survival in hepatocellular carcinoma. *Oncol. Rep.* **30,** 2179–2186 (2013).

9. Huang, A.-H. *et al.* PARVA promotes metastasis by modulating ILK signalling pathway in lung adenocarcinoma. *PloS One* **10,** e0118530 (2015).

10. Steller, E. J. A. *et al.* PDGFRB promotes liver metastasis formation of mesenchymal-like colorectal tumor cells. *Neoplasia N. Y. N* **15,** 204–217 (2013).
